# The ester derivative Palmitoylcarnitine abrogates cervical cancer cell survival by enhancing lipotoxicity and mitochondrial dysfunction

**DOI:** 10.1186/s12964-025-02218-8

**Published:** 2025-05-03

**Authors:** Sangavi Eswaran, Roshan Mascarenhas, Shama Prasada Kabekkodu

**Affiliations:** 1 Department of Cell and Molecular Biology, Manipal School of Life Sciences, Manipal Academy of Higher Education, Manipal, Karnataka 576104 India; 2 Faculty of Medical Sciences, Newcastle University, Newcastle Upon Tyne, NE1 7RU UK; 3 Newcastle University Medicine Malaysia (NUMed), 1, Jalan Sarjana 1, Kota Ilmu, Educity@Iskandar, Iskandar Puteri, Johor 79200 Malaysia

**Keywords:** Cervical cancer, DOC2B, Palmitoyl carnitine, Lipotoxicity, Reactive oxygen species, Mitochondria, Extracellular vesicles, Metabolomics

## Abstract

**Background:**

In cervical cancer (CC), Double C2 Like Domain Beta (DOC2B) functions as a metastatic suppressor. The present study aims to determine whether ectopic expression of DOC2B causes global metabolomic changes in extracellular vesicles (EVs) and corresponds with its tumor suppressive properties.

**Methods:**

Using a retroviral method, we first ectopically expressed DOC2B in SiHa cells, which do not normally express DOC2B.

**Results:**

We observed that ectopically expressed DOC2B significantly altered the global metabolite profile of EVs. Metabolomics identified significant enrichment of palmitoylcarnitine (PC) in EVs upon ectopic expression of DOC2B. We identified that SiHa and HeLa cells exhibited greater cytotoxicity to PC than gingival fibroblast, HaCaT, Cal27, and MCF7. PC treatment reduced the growth, proliferation, and migration of SiHa and HeLa cells, via increasing apoptosis and decreasing S-Phase cells. PC treatment resulted in morphological alterations, decreased length and number of filopodia, and expression of proteins related to cell cycle progression, proliferation, and the epithelial-to-mesenchymal transition. Further, PC treatment caused mitochondrial morphological changes, increased mitochondrial membrane potential, and decreased mtDNA content. The decreased GSH activity, glucose consumption rate, and lactate production upon PC treatment suggest that PC can induce metabolic reprogramming in CC cells. Increased oxidative stress, calcium overload, lipid droplet accumulation, mitochondrial lipotoxicity, and mitophagy suggest that PC can cause mitochondrial dysfunction. N-acetyl cysteine (NAC) treatment reversed the cytotoxic effect of PC, via decreasing lipid peroxidation rate and increasing GSH activity. PC treatment enhanced the cytotoxic effect of cisplatin in CC.

**Conclusion:**

DOC2B restoration or the use of PC may be employed as a novel therapeutic approach for CC.

**Supplementary Information:**

The online version contains supplementary material available at 10.1186/s12964-025-02218-8.

## Introduction

Cervical cancer (CC) is a frequent gynaecological disease that has contributed to 604 000 new cases and 342 000 fatalities in 2020 [1]. India is accountable for 123 907 new cases and 77 348 CC-related deaths annually [2]. Many nations have seen a decline in the incidence of CC because of the effective implementation of HPV vaccinations, Pap tests, and early detection technology [3]. However, many CC cases are discovered at an advanced stage, making treatment challenging. Advanced CC has limited treatment options, showed recurrence, and had a poor survival rate [4, 5]. Hence, it is important to understand the mechanisms contributing to disease onset, progression, and treatment failure.

The human Double C-2-Like domain beta (DOC2B) belongs to the Double C-2 protein family situated at 17q13.3 and has two calcium-binding domains and a Munc interacting domain (MID) [6]. Abnormal expression of DOC2B is reported in Cataract 8 Multiple Types, diabetes, and cancer [7–9]. DOC2B is a hypermethylated and downregulated gene in CC. In CC, the reduced expression of DOC2B is reported in both pre-malignant and malignant conditions [6]. Further, ectopic expression of DOC2B inhibited growth, proliferation, migration, and invasion of CC cell in-vitro. DOC2B overexpression substantially elevated intracellular Ca2 + level which diminished upon its knockdown. Moreover, the presence of DOC2B inhibited in-vivo tumor growth and metastasis. Mechanistically, DOC2B inhibited EMT and promoted senescence in a calcium dependent manner [10]. Another study demonstrated that DOC2B targets Wnt/β-catenin pathway by promoting the proteasomal degradation of CTNNB1 [11]. DOC2B mediated calcium dependent oxidative stress and its association with lipotoxicity and mitochondrial dysfunction is also reported in CC [12]. More recently, we demonstrated that DOC2B is localized to extracellular vesicles (EVs) and EV mediated DOC2B transfer can inhibit tumor growth in CC [13]. Taken together, DOC2B may act as a tumor inhibitory gene in CC.

Extracellular vesicles (EVs) are membrane-bound structures released by the cells during normal and pathological conditions. The cargos present in the EVs play a significant role in intracellular communications [14]. DOC2B is a calcium-dependent protein that regulates exocytosis and membrane trafficking [15, 16]. DOC2B modulates the sorting of cargo into EVs, including lipids and metabolites. A recent study demonstrated that the presence of DOC2B in EVs generated from pancreatic beta cells [17]. Research on DOC2B suggests its potential role in metabolic regulation, notably in glucose homeostasis and adipocyte function [18, 19]. Furthermore, our own studies suggested mitochondrial dysfunction and lipotoxicity by DOC2B in CC cells suggesting its potential role in metabolism [12]. We previously reported the presence of DOC2B in EVs isolated from cervical cancer cell ectopically expressing DOC2B [13]. However, the exact mechanisms by which DOC2B affect the metabolic changes in EV are incompletely understood. In this regard, DOC2B may regulate the sorting of phospholipids, sphingolipids, or carnitine derivatives into EVs, leading to functional metabolic changes. EV metabolomics data revealed that among all identified metabolites, Palmitoyl carnitine (PC) showed high enrichment in DOC2B EVs compared to control EVs. Further, DOC2B EVs nearly 70% metabolites are lipid and its derivatives which makes us more curious to investigate the lipid metabolite in CC.

PC is an ester derivative of carnitine linked with mitochondrial fatty acid oxidation [20]. Malignant melanoma has already been reported to have PC in exosomes [21]. PC treatment induced apoptosis in colorectal cancer, prostate cancer, and liver cancer in-vitro [22–24]. PC exposure increases fatty acid oxidation and glutathione depletion in colorectal cancer cells and affects the mitochondrial respiration rate in liver cancer cells [25, 26]. PC can induce apoptosis by interfering with a few enzymes and transporter proteins on the mitochondrial membrane. PC is reported to enhance the cytotoxic effects of dasatinib in liver cancer [23]. Few studies have shown the potential use of PC against liver cancer, colorectal cancer, and prostate cancer. However, there are no studies that have investigated the biological consequences of PC in CC.

Cisplatin is a drug commonly used to treat CC. But its efficacy is affected by several factors such as intrinsic and acquired resistance, systemic toxicity, and off-target effects, which compromise treatment outcomes [5, 27]. Hence there is need to use approaches that may overcome cisplatin resistance. Earlier studies have shown that the ability of PC to reduce the viability of HT29 and HCT 116 cells [26]. In liver cancer, the combination of PC with Dasatinib has shown synergistic anti-cancer effects [23]. Thus, the present study was undertaken to evaluate the biological functions and significance of PC in CC.

Our results demonstrated that DOC2B overexpression impacted the concentration of metabolites, particularly PC, in EVs. While the previous work provided a foundational understanding of DOC2B’s role in oxidative stress and mitochondrial dysfunction [12], this study extends these findings by elucidating how PC, both in its simple form and within EVs, modulates cancer cell behaviour, particularly in CC models. Our study suggests that PC can promote oxidative stress, intracellular calcium overload, lipotoxicity, mitochondrial dysfunction, and mitophagy in CC cells. Our findings indicate that PC exposure significantly increases the cytotoxic effect of cisplatin, suggesting a possible combinatorial approach to improve therapeutic efficacy.

## Materials and methods

### Cell culture, transfection, and generation of stable cell lines

SiHa and HeLa cell lines procured from the ATCC, USA, were authenticated using STR profiling (Promega, USA). SiHa cells inherently do not express DOC2B. Control and DOC2B overexpression cells were generated by retrovirally transducing SiHa cells using pMXs-IRES-GFP vector as published earlier [6, 10]. We incubated SiHa and HeLa cells with PC (Sigma-Aldrich, USA) concentrations ranging from 5 µM, 10 µM, and 20 µM for 48 h to assess its biological effects. The drugs used for our study as follows: Cisplatin (MedChemExpress, USA), N-Acetyl-L-cysteine (NAC; MP Biomedicals, USA), and Carbonyl cyanide m-chlorophenyl hydrazone (CCCP; MedChemExpress, USA). We used SP8-DMi8 confocal microscope (Leica Microsystems, USA) and LAS X and ImageJ software for all the confocal microscopy imaging and associated image analysis. For biochemical experiments, data was normalized by comparing with total protein unless specified.

### EV isolation and characterization

DOC2B-SiHa and Vector-SiHa cells were cultured in serum-free DMEM for 24 h to obtain a conditioned medium. We utilized the total exosome isolation reagent to extract the EVs from the conditioned medium (Thermo Fisher Scientific, USA). The NanoSight NS300 and Malvern Zetasizer ZS90 (Malvern Instruments, UK) instruments were used to determine the EV count and size distribution. EVs were confirmed by Western blotting for specific markers [28].

### Metabolomics of EVs

EVs collected as above were analysed for changes in global metabolite profile using Agilent 6520 TOF–MS connected to a 1200 series HPLC (Agilent Technologies, USA). We extracted the metabolites by sonicating the EV pellets in pre-chilled 100% methanol. The extracted metabolites are lyophilized and then reconstituted in a solution [water: acetonitrile (95:5 vol/vol) mixture consisting of 0.1% formic acid (HCOOH)] [29]. We separated the metabolites using an Eclipse Plus C18 column. We set the scan range to 50–1700 m/z and the scan rate to 1.4 spectra/sec. The data were analysed using HMDB (www.hmdb.ca/) [30] and MetaboAnalyst-6.0 (www.metaboanalyst.ca/) [31] databases.

### MTT assay

A 96-well plate containing 5 × 10^3^ cells/well was treated with indicated concentration of PC and mitomycin C (10 µg/ml) for 24 h and 48 h. Ten microliters of MTT (5 mg/mL; Sigma-Aldrich, USA) reagent were added to each well. After 4 h, we pipetted supernatant and dissolved the formazan crystals in 100 µL of DMSO. Using a multi-mode reader, we determined the OD at 570/630 nm to estimate the viability of the cells.

### Growth curve analysis

We cultivated 2 × 10^4^ cells in a 3.5-mm cell culture plate and incubated them with PC for 120 h. Trypsin was used to harvest cells at predetermined intervals. A hemocytometer was used to count the number of viable cells. Next, using http://www.doubling-time.com/compute.php, the cell doubling time was computed [10].

### Actin-Phalloidin Staining

Cells cultured on a sterile coverslip were incubated with the indicated concentration of PC for 48 h. Cells received 4% paraformaldehyde treatment for 5 min, washed with PBS, and incubated with 1 µg/mL each of TRITC-conjugated actin-phalloidin (30 min; Sigma‒Aldrich, USA) and Hoechst-33342 (5 min; Sigma-Aldrich, India). We used a 63 × oil immersion objective and an excitation/emission wavelength of 540/570 nm and 340/510 nm, respectively, to capture pictures of the actin-cytoskeletal network and nucleus [32].

### Colony formation assay

We allowed 100 cells to attach to a 35 mm dish overnight and then exposed them to different concentrations of PC. Cells were incubated for 12 days to form colonies. The resulting colonies were stained by applying a 0.5% crystal violet solution prepared in methanol. A microscope was used to count the colonies that had more than 50 cells [13]. The number of colonies in unexposed cells was compared with an exposed group to assess the PC effect on growth.

### Migration assay

Cells at 80–90% confluence were serum starved for 48 h, and a linear cell-free area was created using a sterile micropipette tip. The cells were then exposed to varying concentrations of PC in a complete medium, while no PC was used in the control wells. Cells migrating into the cell-free area were imaged (Olympus, Japan) and analyzed [13].

### Cell cycle analysis

PC exposed and control cells dislodged by trypsinization were fixed by the addition of chilled 70% alcohol and kept at 4 °C overnight. The cell pellets obtained by spinning at 2000 rpm for 10 min were mixed with RNaseA (100 μg/ml, Sigma-Aldrich, USA) and PI (10 μg/mL, Sigma-Aldrich, USA) in the dark. We quantified the cell cycle distribution by a FACS Calibur (B D Biosciences, USA) [10].

### Apoptosis analysis

A 48 h PC exposed and control cells were incubated with 100 μg/ml of acridine orange (AO) and ethidium bromide (EtBr) for 5 min. The photographs of stained cells were captured using a 63 × oil immersion objective using a confocal microscope. The excitation/emission of AO and EtBr used for imaging was 500/526 nm and 482/616 nm, respectively. Morphology and staining patterns were used to score the cells into live, early, and late apoptotic, necrotic, and dead cells [13, 33].

### Mitochondria isolation

As published earlier, the PC-treated and control cells were homogenized using a Dounce homogenizer in lysis buffer [34]. The supernatant collected was spined at 1000 g for 10 min and 15,000 g for 15 min to collect the mitochondrial pellet. The supernatant was collected, washed twice (0.25 M sucrose, 10 mM Hypotonic Tris, pH 7.6), and spun at 16,000 g for 15 min to obtain a pure mitochondrial pellet [35]. All the centrifugation was performed at 4 °C. Western blotting for COX-2 and GAPDH proteins evaluated the purity of mitochondria.

### mtDNA depletion assay

DNA isolated from PC-treated and control cells was used for PCR for mitochondrial-genome-encoded and nuclear-encoded genes. To assess mtDNA depletion, the ratio of mtCOX-2 (mitochondrial genome encoded gene) to that of ACTB (nuclear-encoded gene) by densitometric analysis was used [36]. DNA was extracted using a commercially available DNA extraction kit (Sigma-Aldric, USA) and quantified using spectrophotometry. PCR was carried out for mtCOX2 (Forward: CCGACTACGGCGGACTAATC; Reverse: CGCCTGGTTCTAGGAATAATGG) and ACTB (Forward: GACGACATGGAGAAAATCTG; Reverse: ATGATCTGGGTCATCTTCTC) (Supplementary Table 1). A UV gel documentation system was used to image the PCR products after they had been resolved in a 2% agarose gel. The intensity of the PCR product was directly proportional to the quantity of mtDNA. The data was represented as the ratio between mtDNA vs. nuclear DNA intensity.

### Semiquantitative reverse transcriptase PCR

We employed RT-PCR to determine the expression of *PGC1 A, TFB1M, COX2, SIRT3, NRF1, MFN1, and MFN2*. TRIzol reagent (Life Technologies, USA) was used to extract RNA from PC-exposed and control cells. The cDNA generated using a High-Capacity cDNA Archive Kit (Thermo Fisher Scientific, USA) was subjected to gene-specific PCR in a Veriti Thermocycler (Applied Biosystems, USA). The details regarding primer sequences, annealing temperatures, and PCR amplicon sizes are provided in Supplementary Table 1. The relative gene expression was determined using densitometric analysis of PCR product resolved in an ethidium bromide-stained agarose gel with NIH ImageJ software employing β-Actin (*ACTB*) as the internal control.

### Western blotting

PC-exposed and control cells were lysed in RIPA buffer and quantified using a BCA kit from Sigma, USA. Equal concentrations of total proteins after separation in an SDS-PAGE were transferred to a nitrocellulose membrane (GE Healthcare, USA) and immersed in 5% non-fat dry milk or BSA. The list of 1º antibodies used in the study was against DOC2B, Caspase 9, Cleaved-Caspase 3 (Proteintech, USA), pERK1/2, ERK1/2, pAKT, AKT, CCND1, CDH1, CDH2, cMyc, SNAI1, SLUG, VIM, TFAM, GAPDH (Cell Signaling Technologies, USA), MFN1, CPT1 A, PDK3, LC3B, β-Actin (ABclonal, USA), CD9, PGC1 alpha, SIRT3, COX2 (ThermoFisher Scientific, USA), CD63, BECN1 (Cloud Clone Corp., USA), and CCNE (Santa Cruz Technologies, USA) (Table 1). TBST-washed membranes were probed with rabbit or mouse HRP conjugated secondary antibodies (Cell Signaling Technologies, USA) for 2 h at room temperature. Blots were developed using SuperSignal West Pico chemiluminescent substrate (Thermo Fisher Scientific, USA) and imaged using an Image Quant LAS 4000 (GE Healthcare, USA) [32].

**Table 1:** List of primary and secondary antibodies

Antibody Name	Isotype	Working Dilutions	Manufacturer	Catalogue Number
DOC2B	Rabbit	1:5000	Proteintech, USA	20,574–1-AP
Caspase 9	Mouse	1:7500	Proteintech, USA	66,169–1-Ig
Cleaved- caspase 3	Rabbit	1:5000	Proteintech, USA	25,128–1-AP
CD63	Rabbit	1:1000	Cloud Clone Corp., USA	PAB345Hu01
CD9	Rabbit	1:1000	ThermoFisher Scientific, USA	MA5-31,980
pERK1/2	Rabbit	1:3000	Cell Signaling Technologies, USA	4370S
ERK1/2	Rabbit	1:3000	Cell Signaling Technologies, USA	4695S
pAKT	Rabbit	1:3000	Cell Signaling Technologies, USA	4060S
AKT	Rabbit	1:3000	Cell Signaling Technologies, USA	4691S
Cyclin D1	Rabbit	1:3000	Cell Signaling Technologies, USA	2978 T
Cyclin E	Mouse	1:3000	Santa Cruz Technologies, USA	sc-247
CDH1	Rabbit	1:3000	Cell Signaling Technologies, USA	3195 T
CDH2	Rabbit	1:3000	Cell Signaling Technologies, USA	13116 T
cMyc	Rabbit	1:3000	Cell Signaling Technologies, USA	5605 T
SNAI1	Rabbit	1:3000	Cell Signaling Technologies, USA	3879 T
SLUG	Rabbit	1:3000	Cell Signaling Technologies, USA	9585 T
VIM	Rabbit	1:3000	Cell Signaling Technologies, USA	5741 T
PGC1 alpha	Rabbit	1:3000	ThermoFisher Scientific, USA	PA5-72,948
SIRT3	Rabbit	1:3000	ThermoFisher Scientific, USA	MA5-14,910
TFAM	Rabbit	1:3000	Cell Signaling Technologies, USA	7495
COX2	Mouse	1:3000	ThermoFisher Scientific, USA	A-6404
MFN1	Rabbit	1:3000	ABclonal, USA	A9880
CPT1 A	Rabbit	1:3000	ABclonal, USA	A5307
PDK3	Rabbit	1:3000	ABclonal, USA	A8028
BECN1	Rabbit	1:2000	Cloud Clone Corp., USA	PAJ557Hu01
LC3B	Rabbit	1:3000	ABclonal, USA	A19665
GAPDH	Rabbit	1:3000	Cell Signaling Technologies, USA	2118
β-Actin	Rabbit	1:10,000	ABclonal, USA	AC026
anti-rabbit IgG-HRP	Rabbit	1:5000	Cell Signaling Technologies, USA	7074S
anti-mouse IgG-HRP	Mouse	1:5000	Cell Signaling Technologies, USA	7076S

### Nile red staining

PC-treated and control cells, upon fixing with 4% paraformaldehyde for 5 min, were immersed first in 5 μg/mL of Nile Red (ThermoFisher Scientific, USA) and then with 1 μg/mL of Hoechst-33342 (Sigma Aldrich USA). For localization of lipid droplets in mitochondria, serum-starved cells were exposed to PC and subjected to staining with 5 μg/mL of Rhodamine123 for 30 min, 5 μg/mL of Nile Red and 1 μg/mL of Hoechst-33342 for 10 min in dark. The excitation/emission peaks of Rhodamine123, Nile Red, and Hoechst-33342 were 508 nm/528 nm, 552 nm/636 nm, and 361 nm/497 nm, respectively [12]. The fluorescent images were taken at 63 × oil immersion objective in a confocal microscope. Colocalization and Pearson correlation analysis between Rhodamine 123 and Nile red stained region was conducted to identify the lipid droplet within mitochondria.

### Estimation of lipid peroxidation rate

The PC exposed and control cells were suspended in cell lysis buffer (20 mM Tris–HCl pH 7.5, 150 mM NaCl, 1 mM EDTA, 1% Triton X-100) and treated with 20% TCA. The supernatant collected by centrifugation was incubated with 1% thiobarbituric acid in a boiling water bath for 30 min. A multimode reader set to 532 nm was used to quantify the generated colour [12].

### Confocal microscopy

The PC exposed and control cells received treatment for 30 min at 37 °C in the dark with 5 µM of Rhodamine 123 (Mitochondrial membrane potential; 508 nm/528 nm), DCFDA (ROS; 485 nm/535 nm), Mitosox-Red (Mitochondrial ROS; 396 nm/610 nm), Nonyl acridine orange (NAO, Mitochondrial Cardiolipin; 496 nm/519 nm), 50 nM MitoTracker Red (Mitochondrial morphology; 581 nm/644 nm) in a CO_2_ incubator. Mitochondrial morphology was analyzed using ImageJ. The cells were stained with 1 µM each of Fluo-3 AM (excitation: 506 nm and emission: 526 nm) and Rhod-2 AM (excitation: 552 nm and emission: 581 nm) separately in HBSS containing 1 mg/ml of glucose for 45 min at 37 °C. The mean fluorescence intensity of 100 cells from 5 randomly selected fields acquired using a confocal microscope was computed using LAS X software.

### ATP estimation

We used an ATP determination kit (Life Technologies, USA) and a luminometer to quantify the changes in cellular ATP content in response to PC exposure, as published earlier [12].

### Glucose uptake assay

We quantified the changes in glucose uptake rate in response to PC treatment using a glucose assay kit (Agappe, India) in a multimode reader. The glucose uptake rate was estimated by subtracting the glucose remaining in the conditioned medium at the indicated time point from the original glucose level in the media.

### Lactate measurement

The PC exposed and control cells were incubated with lysis buffer (20 mM Tris–HCl pH 7.5, 150 mM NaCl, 1 mM EDTA, 1% Triton X-100). We added 50 mM PIPES with pH 7.5 to the supernatant and kept it for 10 min. The colour developed were recorded at 505 nm by employing a multimode reader.

### GSH estimation

We combined cell lysate with 5% sulfosalicylic acid, kept it at 4 °C for 1 h, and spun at 12,000 rpm at 4 °C for 10 min. We combined 100ul of supernatant with 894ul of 0.1 M potassium phosphate buffer pH 7.4 and 6 µl of 5.5 dithiobis-(2,-nitro benzoic acid) pH 8.0. Color formed after 30 min of incubation at 37 °C was recorded at 412 nm by a spectrophotometer [37].

### Statistical analysis

We performed the experiments two times in triplicates. Findings from experiments were reported as mean. A one-way ANOVA and a two-tailed Student’s t-test were used to compare the results. A p < 0.05, which was considered statistically significant.

## Result

### DOC2B significantly alters the metabolite distribution in EVs from SiHa cells

**Fig. 1:**
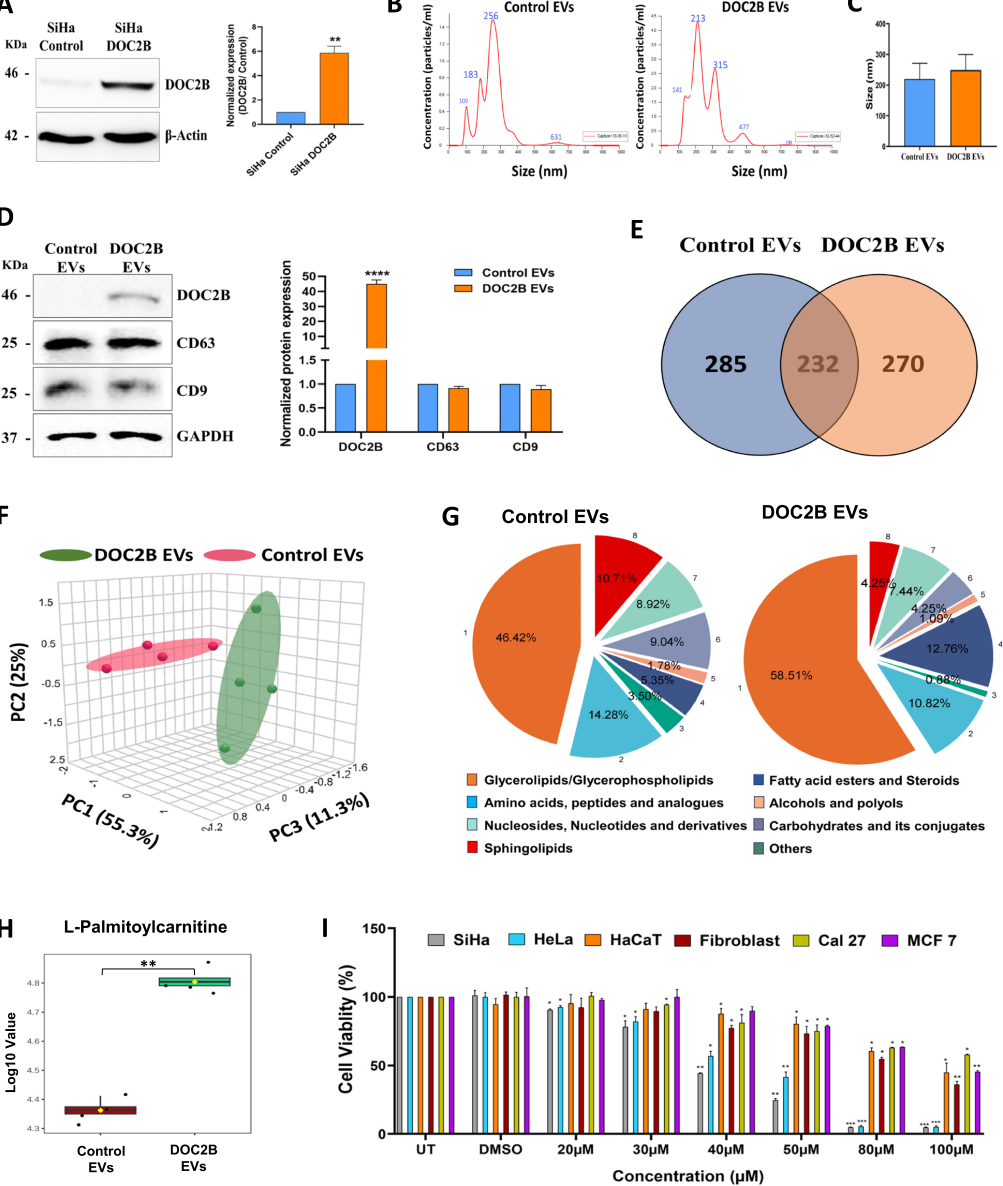
Ectopic expression of DOC2B in SiHa alters the global metabolite profile of EVs. **A** Representative Western blot image and bar graph confirming the ectopic expression of DOC2B in SiHa cells. **B** The EV size distribution was measured by nanoparticle tracking analysis (NTA). **C** Size distribution of EVs collected from Vector transfected (Control) and DOC2B transfected (DOC2B) SiHa cells. **D** Western blotting analysis of EVs collected from Vector transfected and DOC2B transfected SiHa cells and probed for CD63, CD9, and DOC2B. **E** The Venn diagram represents common and unique metabolites identified by LC–MS analysis of EVs. **F** Principal component analysis (PCA) performed using EV metabolites from Vector transfected and DOC2B transfected SiHa cells. **G** The metabolite distribution in EVs collected from Vector transfected and DOC2B transfected SiHa cells. **H** The metabolite Palmitoylcarnitine (PC) abundance in control and DOC2B EVs. In DOC2B EVs, the PC level is significantly increased compared to control EVs. **I** Cytotoxic effects of PC on various cell lines. The bar graph represents the percentage cell viability of SiHa, HeLa, Fibroblast, HaCaT, Cal27, and MCF7 cells in response to PC exposure for 48 h as analyzed by MTT assay. Data presented are mean ± SD of three independent experiments in triplicate (Mito C-Mitomycin C (10 µg/ml)). *P < 0.05, **P < 0.01, and ***P < 0.001 indicates statistical significance

A global metabolomic analysis was carried out to determine the consequences of DOC2B manipulation on the metabolomic profile of EVs and to understand its biological consequences in recipient cells. EVs were isolated from DOC2B overexpressed SiHa (Fig. 1A) and subjected to NTA and Zetasizer to measure its size (Fig. 1B, C, and Supplementary Fig. 1 A) and Western Blot to confirm EVs surface markers (Fig. 1D). DOC2B re-expression significantly altered the metabolite composition in the EVs. The total number of metabolites identified was 517 and 502 in EVs isolated from control-SiHa and DOC2B-SiHa cells (Fig. 1E). PCA analysis of metabolite profiles from EVs displayed a clear separation between control-SiHa and DOC2B-SiHa (Fig. 1F). The distribution of amino acids, peptides and analogues, carbohydrates and carbohydrate conjugates, nucleoside and nucleoside derivatives, sphingolipids, and alcohols and polyols distribution were lower in EVs harvested from DOC2B-SiHa cells than control-SiHa EVs. However, the distribution of glycerolipids/glycerophospholipids, fatty acid esters, and steroids increased in EVs collected from DOC2B-SiHa cells than control-SiHa EVs (Fig. 1G). Table 2 shows the list of identified metabolites in control-SiHa and DOC2B-SiHa EVs. The PATHVIEW [38] image shows the DOC2B EVs enriched glycerophospholipid metabolism (Supplementary Fig. 2). The top 20 pathways enriched between control-SiHa and DOC2B-SiHa are shown in Supplementary Fig. 3. KEGG pathways provide a comprehensive, visual, and functional representation of molecular networks that help in understanding complex biological systems. The pathways such as pantothenate and COA synthesis, phosphatidylethanolamine and phosphatidylcholine biosynthesis, sphingolipid metabolism, and fatty acid metabolism are highly enriched KEGG pathways in DOC2B EVs compared to control EVs. It is evident that the metabolites of DOC2B EVs are enriched with lipids and fatty acid derivatives, which are responsible for executing lipid biosynthesis. Collectively, our data showed that DOC2B re-expression significantly altered the metabolome of EVs. Palmitoylcarnitine is one of the metabolites whose abundance was significantly altered in EVs in response to DOC2B re-expression (Fig. 1H). Hence, we investigated the biological significance of PC in CC via cell and biochemical experiments.

**Table 2:** List of identified metabolites

Compound Name	m/z	Monoisotopic mass	Adduct	HMDB ID	Formula	Fold Change (DOC2B/Control)−1
Adenosine 3’,5’-diphosphate	428.0379	427.0294147	M + H	HMDB0000061	C10H15 N5O10P2	0.023
Adenosine tetraphosphate	587.9702	586.9620756	M + H	HMDB0001364	C10H17 N5O16P4	−0.017
Cardiolipin(22:5/22:5/22:5/22:5)	1650.04967	1649.034826	M + H	HMDB0059480	C97H150O17P2	0.211
CE(24:1(15Z))	735.7089	734.6940821	M + H	HMDB0006728	C51H90O2	0.186
Ceramide (d18:0/14:0)	512.5145	511.496445	M + H	HMDB0011759	C32H65 NO3	0.061
Ceramide (d18:1/20:0)	594.5888	593.5746953	M + H	HMDB0004951	C38H75 NO3	0.163
Cytidine 5’-triphosphate	483.9987878	482.9845118	M + H	HMDB0000082	C9H16 N3O14P3	−0.084
Deoxyadenosine	252.1113	251.1018393	M + H	HMDB0000101	C10H13 N5O3	−0.069
Deoxyinosine	253.0994	252.0858549	M + H	HMDB0000071	C10H12 N4O4	−0.079
Dephospho-CoA	688.1578	687.148878	M + H	HMDB0001373	C21H35 N7O13P2S	−0.056
DG(16:0/16:0)	569.52134	568.5066753	M + H	HMDB0007098	C35H68O5	−0.028
Diacylglycerol	595.5349	594.5223254	M + H	HMDB0242173	C37H70O5	−0.089
Dihydrocortisol	365.2399	364.2249741	M + H	HMDB0003259	C21H32O5	0.080
Dodecanedioic acid	231.1590852	230.1518092	M + H	HMDB0000623	C12H22O4	0.041
dUMP	309.04987	308.0409519	M + H	HMDB0001409	C9H13 N2O8P	0.068
Gamma-Glutamylalanine	219.09978	218.0902716	M + H	HMDB0006248	C8H14 N2O5	0.039
Gamma-glutamylmethionine	279.1029	278.0936429	M + H	HMDB0034367	C10H18 N2O5S	−0.124
Glycerol 3-phosphate	173.0219	172.0136745	M + H	HMDB0000126	C3H9O6P	−0.231
Glycerophosphocholine	258.115	257.1028244	M + H	HMDB0000086	C8H20 NO6P	−0.031
Inosinic acid	349.0578	348.0470999	M + H	HMDB0000175	C10H13 N4O8P	−0.102
Isoglobotriaose	503.1981	502.1897704	M + H	HMDB0006598	C19H34O15	−0.007
L-Palmitoylcarnitine	400.3423	399.3348589	M + H	HMDB0240774	C23H45 NO4	0.0135
L-Proline	116.0723	115.0633285	M + H	HMDB0000162	C5H9 NO2	−0.005
LysoPA (i-20:0/0:0)	510.3589	509.3481395	M + H	HMDB0011511	C25H52 NO7P	0.110
LysoPA(0:0/16:0)	411.2549	410.2433401	M + H	HMDB0007849	C19H39O7P	−0.061
LysoPC (14:1(9Z)/0:0)	466.2939	465.2855393	M + H	HMDB0010380	C22H44 NO7P	0.324
Lysophosphatidic acid (16:0)	411.257	410.2433401	M + H	HMDB0007853	C19H39O7P	0.041
Lysophosphatidylcholine (26:1)	634.4825	633.47334	M + H	HMDB0029220	C34H68 NO7P	0.097
Lysophosphatidylethanolamine (18:2)/(0:0)	478.2949	477.2855393	M + H	HMDB0011507	C23H44 NO7P	0.190
N-Acetyllactosamine	384.151	383.1427606	M + H	HMDB0001542	C14H25 NO11	0.061
N-Acetyl-L-tyrosine	224.095	223.0844579	M + H	HMDB0000866	C11H13 NO4	0.046
Octaprenyl diphosphate	723.455	722.4440276	M + H	HMDB0001094	C40H68O7P2	−0.005
PA (8:0/18:0)	565.3898	564.3791058	M + H	HMDB0115489	C29H57O8P	0.175
PA(14:1(9Z)/14:0)	591.4098	590.3947559	M + H	HMDB0114795	C31H59O8P	0.160
PC(O-22:0/18:3(6Z,9Z,12Z))	840.6407	839.6404054	M + H	HMDB0008183	C48H90 NO8P	0.166
PE(20:0/14:1(9Z))	718.5311	717.5308549	M + H	HMDB0009218	C39H76 NO8P	0.060
PE(20:4(5Z,8Z,11Z,14Z)/P-16:0)	740.5225	739.5152049	M + H	HMDB0009385	C41H74 NO8P	−0.011
PG(20:3(5Z,8Z,11Z)/18:2(9Z,12Z))	797.521	796.5254352	M + H	HMDB0010654	C44H77O10P	−0.131
PGP(22:6(4Z,7Z,10Z,13Z,16Z,19Z)/ 20:4(8Z,11Z,14Z,17Z))	923.4788	922.4761165	M + H	HMDB0116479	C48H76O13P2	−0.034
PGP(i-13:0/i-19:0)	803.4701	802.4761165	M + H	HMDB0116561	C38H76O13P2	−0.012
Phenol glucuronide	271.0834	270.0739528	M + H	HMDB0060014	C12H14O7	−0.029
Phenylalanine	166.0898	165.0789786	M + H	HMDB0000159	C9H11 NO2	0.072
Phosphatidic acid (22:6(4Z,7Z,10Z,13Z,16Z,19Z)/ 20:4(5Z,8Z,11Z,14Z))	769.4812	768.4730062	M + H	HMDB0115416	C45H69O8P	0.085
Phosphatidylcholine (16:1(9Z)/22:0)	816.6416	815.6404054	M + H	HMDB0008018	C46H90 NO8P	0.135
Phosphatidylcholine (18:4(6Z,9Z,12Z,15Z)/18:4(6Z,9Z,12Z,15Z))	774.5001	773.4995548	M + H	HMDB0008240	C44H72 NO8P	−0.075
Phosphatidylethanolamine (P-18:0/20:4(5Z,8Z,11Z,14Z))	768.5597	767.546505	M + H	HMDB0009003	C43H78 NO8P	0.036
Phosphatidylglycerol (i-12:0/i-12:0)	611.3998	610.3845851	M + H	HMDB0116660	C30H59O10P	−0.131
Phosphatidylglycerol phosphate (i-12:0/18:2(9Z,11Z))	771.4219	770.4135162	M + H	HMDB0116530	C36H68O13P2	−0.039
Phosphatidylglycerol phosphate (i-12:0/i-16:0)	747.4257	746.4135162	M + H	HMDB0116540	C34H68O13P2	−0.048
Phosphatidylserine	386.1215	385.1137825	M + H	HMDB0014291	C13H24 NO10P	0.002
Phosphatidylserine (14:1(9Z)/22:6(4Z,7Z,10Z,13Z,16Z,19Z))	778.4623	777.4580839	M + H	HMDB0012351	C42H68 NO10P	0.018
Phosphatidylserine(20:5 (5Z,8Z,11Z,14Z,17Z)/15:0)	768.485	767.4737345	M + H	HMDB0112681	C41H70 NO10P	−0.002
Phosphoribosyl-ATP	720.01161	719.0043343	M + H	HMDB0003665	C15H25 N5O20P4	0.041
PIP2(18:1(9Z)/18:1(11Z))	1023.4873	1022.48979	M + H	HMDB0010076	C45H85O19P3	0.085
PIP2(20:4(8Z,11Z,14Z,17Z)/18:1(9Z))	1045.4817	1044.47414	M + H	HMDB0010139	C47H83O19P3	0.072
PS(14:1(9Z)/18:4(6Z,9Z,12Z,15Z))	726.431	725.4267843	M + H	HMDB0112309	C38H64 NO10P	0.018
PS(18:0/18:2(9Z,12Z))	788.5492	787.5363342	M + H	HMDB0012380	C42H78 NO10P	0.053
Ribose 1,5-bisphosphate	310.99276	309.9854843	M + H	HMDB0011688	C5H12O11P2	−0.028
Sphingomyelin (d18:1/12:0)	647.5195	646.5049745	M + H	HMDB0012096	C35H71 N2O6P	0.165
TG(12:0/15:0/22:0)	821.7599	820.7519909	M + H	HMDB0095502	C52H100O6	0.210
TG(15:0/15:0/15:0)	765.71	764.6893907	M + H	HMDB0042989	C48H92O6	0.022
TG(20:0/16:0/18:0)	891.8355	890.8302413	M + H	HMDB0063060	C57H110O6	−0.014
Ubiquinone-2	319.1911	318.1831093	M + H	HMDB0006709	C19H26O4	−0.068
(S)−2-hydroxyoctadecanoic acid	301.2749	300.266445	M + H	HMDB0242145	C18H36O3	0.011
2-Phenylaminoadenosine	359.1489	358.1389531	M + H	HMDB0001069	C16H18 N6O4	−0.118

### Exposure to PCs reduces cell proliferation and increases cytotoxicity

We tested the effect of PC on SiHa and HeLa (cervical cancer), gingival fibroblast, HaCaT (immortalized keratinocytes), Cal27 (oral cancer), and MCF7 (breast cancer) cell proliferation by MTT assay (Fig. 1I and Supplementary Fig. 1B). Treatment with 1 µM to 10 µM of PC for 48 h showed no significant cytotoxicity in the cell lines tested. Exposure to 20 µM to 200 µM of PC for 48 h showed varying degrees of cytotoxicity in cell lines tested (Supplementary Fig. 1B). Further, PC treatment with 30 µM to 200 µM showed the highest cytotoxicity in SiHa and HeLa cells. The IC50 value was 33.3 µM, 42.9 µM, 94.1 µM, 82.7 µM, 151.1 µM, and 94.2 µM for SiHa, HeLa, HaCaT, gingival fibroblast, Cal27, and MCF7, respectively (Fig. 1I). Further, no significant cytotoxicity was observed in PC treatment at various concentrations (≤ 30 µM) after 24 h in CC cells (Supplementary Fig. 1 C). Our data suggested that CC cells were more cytotoxic to PC than other normal and cancer cell lines tested.

### PC reduces the colony-forming ability of CC cells

**Fig. 2:**
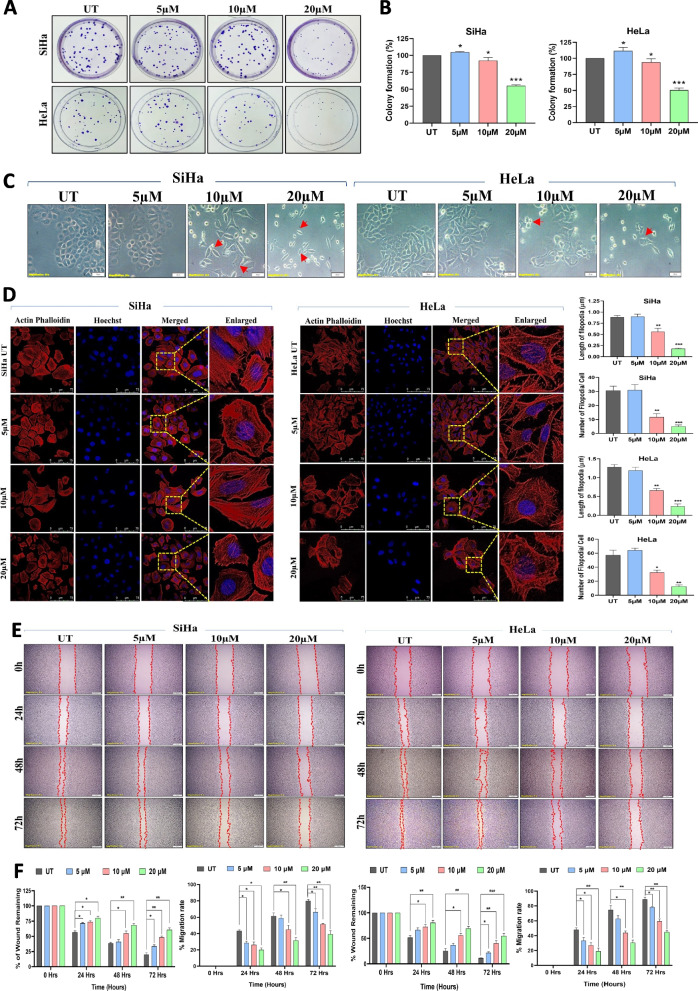
PC treatment reduces growth, migration and induces morphological changes in CC cells. **A** Representative images of crystal violet stained SiHa and Hela cells exposed to PC. Untreated cells served as control. PC treatment significantly reduced the colony-forming ability of SiHa and HeLa cells. **B** Bar graph representing the percentage colony number in PC-exposed and control SiHa and HeLa cells. **C** Morphology of SiHa and Hela cells exposed to indicated concentrations of PC for 48 h (Red arrow indicates a change in cell morphology (spindle shape and chromatin condensation)). **D** Actin-phalloidin and Hoechst stained SiHa and HeLa cells treated with PC imaged using a confocal microscope. The bar graph represents the quantitative data for the length and number of filopodia per cell. **E** Representative image of cell migration assay. **F** Bar graph showing a significant reduction in cell migration upon PC exposure in SiHa and HeLa cells. *P < 0.05, **P < 0.01, and ***P < 0.001 indicates statistical significance

We tested the capacity of PC to inhibit SiHa and HeLa cells to form colonies using a colony formation assay. There was a significant decline in the number of colonies generated upon exposure with 10 µM and 20 µM of PC (Fig. 2A). Treatment with 20 µM of PC showed approximately 50% reduction in colony numbers than untreated controls (Fig. 2B).

### PC induces morphological changes

The impact of PC exposure on CC cell morphology was evaluated by actin-phalloidin staining and confocal microscopy. Bright-field microscopy images showed that exposure to 10 µM and 20 µM of PC showed visible morphological changes in SiHa and HeLa cells (Fig. 2C). PC induces morphological changes including cell shrinkage, chromatin condensation, and membrane blebbing were observed in both cell lines. PC exposure changed the actin cytoskeletal arrangements in SiHa and HeLa cells more than controls (Fig. 2D).

### PC inhibits migration and induces apoptosis, cell cycle arrest of CC cells

**Fig. 3:**
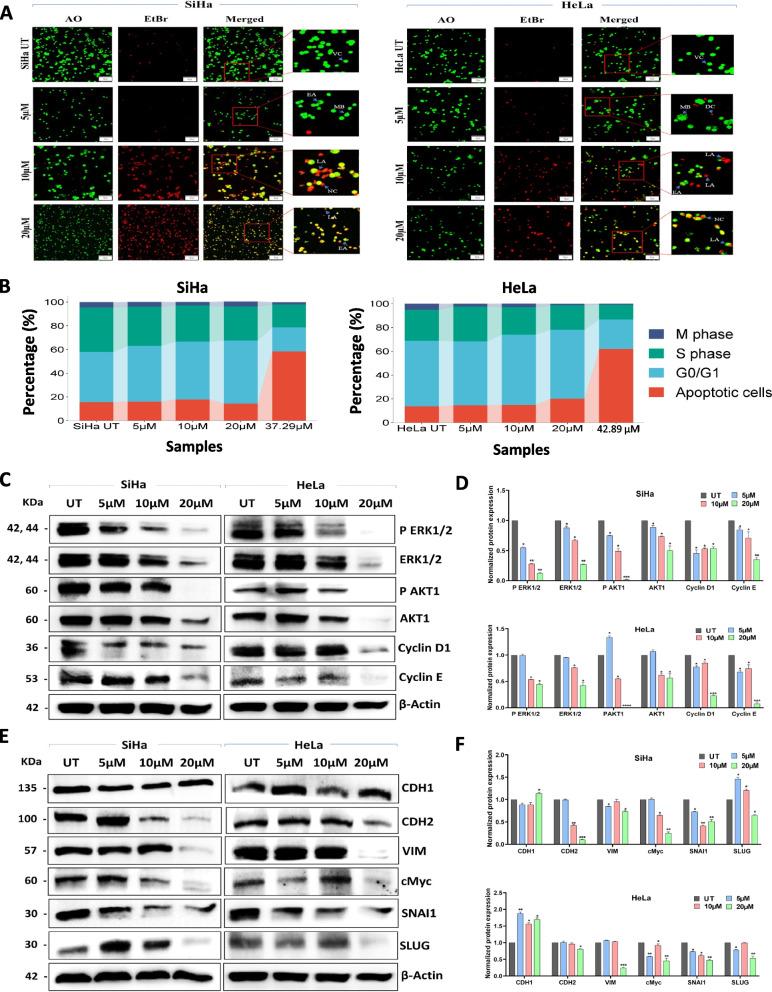
PC induces apoptosis, cell cycle arrest and reduces the expression of genes linked to cell proliferation and EMT. **A** Confocal images of AO/EtBr stained SiHa and HeLa cells before and after PC exposure. **B** Bar graph representing the cell cycle analysis of PC exposed and control SiHa and HeLa cells. **C** Representative Western blot images showing the reduction of genes associated with cell proliferation (AKT1 and ERK1/2) and cell cycle (Cyclin D1 and Cyclin E) in SiHa and HeLa treated with indicated concentrations of PC. **D** The bar graph represents the results of densitometric analysis of western blot images analyzed using ImageJ software. The data was normalized by comparing it with β-Actin. **E** Representative Western blot images showing the upregulation of CDH1 and downregulation of CDH2, VIM, c-Myc, SNAI1, and SLUG in SiHa and HeLa cells treated with PC. **F** Bar graph showing the quantitative analysis of CDH1, CDH2, VIM, c-Myc, SNAI1, and SLUG expression in PC-treated SiHa and HeLa cells. *P < 0.05, **P < 0.01, and ***P < 0.001 indicates statistical significance

Using the scratch assay, PC’s ability to prevent SiHa and HeLa cell migration was evaluated. Following a 72-h incubation period, SiHa and HeLa cells treated with 5 µM, 10 µM, and 20 µM of PC exhibit lesser cell migration into the injured area than control cells. (Fig. 2E and F). According to AO/EtBr staining and confocal microscopy, PC treatment reduced the percentage of viable cells with an associated increase in apoptotic cells (Fig. 3A and Supplementary Fig. 4 A). PC treated significantly increased the percentage of PI-positive cells in both SiHa (58.48% vs. 15.62%) and HeLa (61.9% vs. 13.82%) cells than respective control cells as evaluated by flow cytometry. Simultaneously, PC treatment significantly reduced the % of S-phase cells in both SiHa (19.27% vs. 37.52%) and HeLa (12.36% vs. 25.96%) cells than respective control cells (Fig. 3B and Supplementary Fig. 4B).

### PC exposure reduces proliferation and EMT pathway gene expression

The amount of Cyclin D1, Cyclin E, and the active forms of ERK1/2 and AKT were significantly reduced in SiHa and HeLa cells after 48 h of PC treatment (Fig. 3C). Moreover, 20 µM of PC treatment reduced total ERK1/2 and AKT levels in both cell lines (Fig. 3D). Among the EMT markers, the CDH2, VIM, SNAI1, SLUG, and cMyc levels were downregulated in both cell lines exposed to 20 µM of PC. Treatment with 10 µM of PC reduced CDH2, SNAI1, and cMyc levels while slightly elevated SLUG expression in SiHa. HeLa cells incubated with 10 µM of PC showed downregulation of CDH1 and SNAI1 with simultaneous elevation of cMyc (Fig. 3E). SiHa cells treated with 5 µM of PC showed downregulation of SNAI1, upregulation of SLUG, and no change in cMyc and VIM. HeLa cells treated with 5 µM of PC displayed elevation in CDH1, downregulation of cMyc, SNAI1, SLUG, and no changes in expression of CDH2 and VIM. The epithelial marker, CDH1 was increased at 20 µM, with no difference observed at 5 µM and 10 µM in SiHa cells. However, a significant increase in CDH1 was observed in HeLa cells (Fig. 3F). Thus, treatment with 20 µM PC downregulated the expression of EMT pathway genes.

### PC treatment induces mitochondrial dysfunction and mitochondrial lipotoxicity

**Fig. 4:**
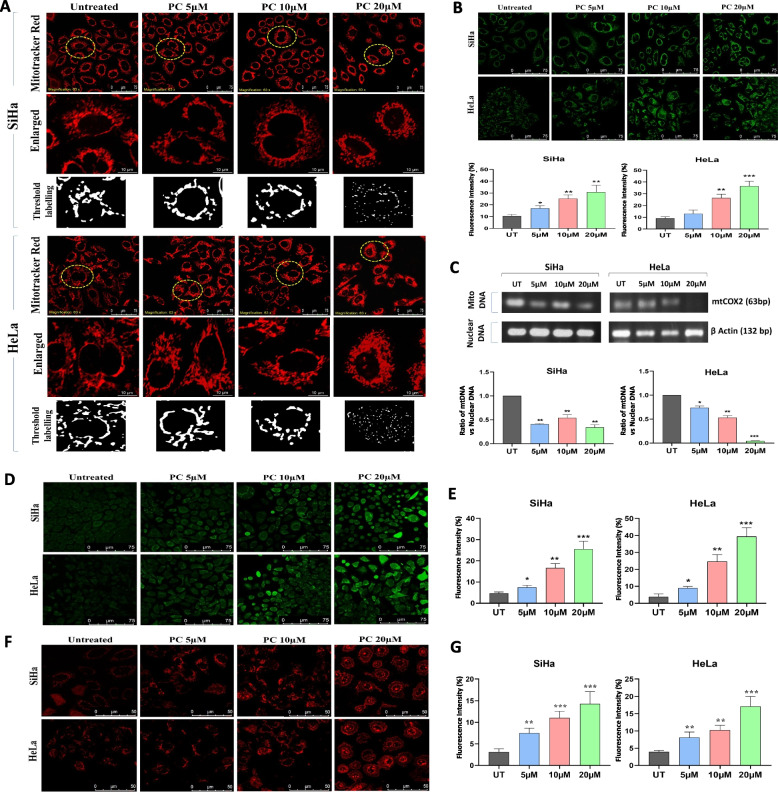
PC treatment induces mitochondrial dysfunction, ROS and inhibits mtDNA in CC. **A** Representative confocal microscopy images showing the changes in mitochondrial morphology and its distribution in PC-treated SiHa and HeLa cells. **B** Representative confocal microscopy images of PC-treated SiHa and HeLa cells stained with Rhodamine-123. Bar graphs illustrate the dose-dependent elevation in MMP in PC-treated SiHa and HeLa. **C** The bar graph represents the mtDNA content analysis. The bar graph represents the ratio between mtCOX and nuclear β-Actin. **D** Represents the DCFDA stained control and PC exposed SiHa and HeLa cells. **E** The bar graph represents the mean fluorescence intensity of the DCFDA in control and PC-exposed SiHa and HeLa cells. **F** Represents the mitoSOX stained control and PC-exposed SiHa and HeLa cells. **G** The bar graph represents the mean fluorescence intensity of the mitoSOX in control and PC-exposed SiHa and HeLa cells. *P < 0.05, **P < 0.01, and ***P < 0.001 indicates statistical significance

**Fig. 5:**
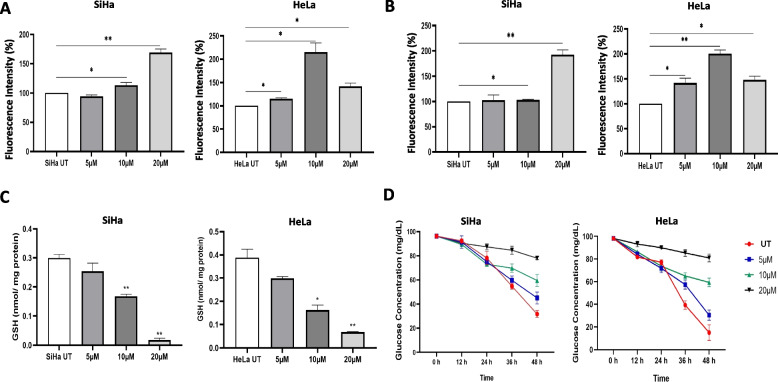
PC affects intracellular and mitochondrial calcium in CC cells. **A** The bar graph represents the quantification of intracellular Ca^2+^ levels in SiHa and HeLa cells exposed to PC. **B** The bar graph quantifies mitochondrial Ca^2+^ levels in SiHa and HeLa cells exposed to PC. **C** The bar graph represents the GSH level in control and PC-exposed SiHa and HeLa cells. **D** The line graphs represent the glucose uptake rate in control and PC-exposed SiHa and HeLa cells. The biochemical assay data were normalized using total protein. *P < 0.05, **P < 0.01, and ***P < 0.001 indicates statistical significance

Metabolomics data suggested that the abundance of fatty acids and lipids are the most changed metabolites in EVs in response to DOC2B overexpression. Excessive lipid metabolism and ROS generation may bring about changes in mitochondrial structure and function. PC treated cells were stained with MitoTracker Red, and mitochondrial structural parameters were analyzed using ImageJ. Control cells showed well-developed and interconnected mitochondrial networks. However, treatment with PC reduced the mitochondrial interconnections and changed the filamentous mitochondria to a circular shape in both cell lines (Fig. 4A and Supplementary Fig. 5). Mitochondrial morphology is a critical factor that determines cell fate. Fragmentation relates to cell death, while fusion is reported to enhance survival. During apoptosis, mitochondria undergo fragmentation releasing the pro-apoptotic factors including cytochrome c release and caspase activation [39]. Moreover, damaged spherical mitochondria are selectively targeted for mitophagy via the PINK1/Parkin pathway [40]. Thus, mitochondrial shape transitions act as critical determinants of apoptosis and mitophagy, reflecting the cell’s metabolic and survival status. We observed an increase in Mitochondrial Mass (Supplementary Fig. 6 A), MMP (Fig. 4B), and a decrease in mtDNA in response to PC treatment (Fig. 4C). A dose-dependent increase in total and mitochondrial ROS levels (Fig. 4D, E, F and G). PC treatment increased intracellular and mitochondrial calcium levels in HeLa cells compared to unexposed control cells (Fig. 5A). However, SiHa cells displayed an increase in intracellular and mitochondrial calcium only at 20 µM of PC (Fig. 5B). and a dose-dependent decrease in GSH level upon PC exposure were also noted (Fig. 5C).

We performed biochemical experiments to measure the glucose uptake rate, intracellular ATP levels, and lactate production to assess the impact of PC on metabolism. A dose-dependent decrease in glucose uptake was observed with the increase in PC dose in both the CC cell lines (Fig. 5D). Incubation with 10 µM and 20 µM of PC showed a significant decrease in lactate levels in both cell lines (Supplementary Fig. 6B). Incubation with 5 µM of PC showed a higher intracellular ATP level in both cell lines. When PC was applied at concentrations of 10 µM and 20 µM, HeLa cells showed a noticeably lower level of intracellular ATP. However, 10 µM PC treatment significantly increased intracellular ATP in SiHa cells, while 20 µM PC treatment had no appreciable impact (Supplementary Fig. 6 C).

**Fig. 6:**
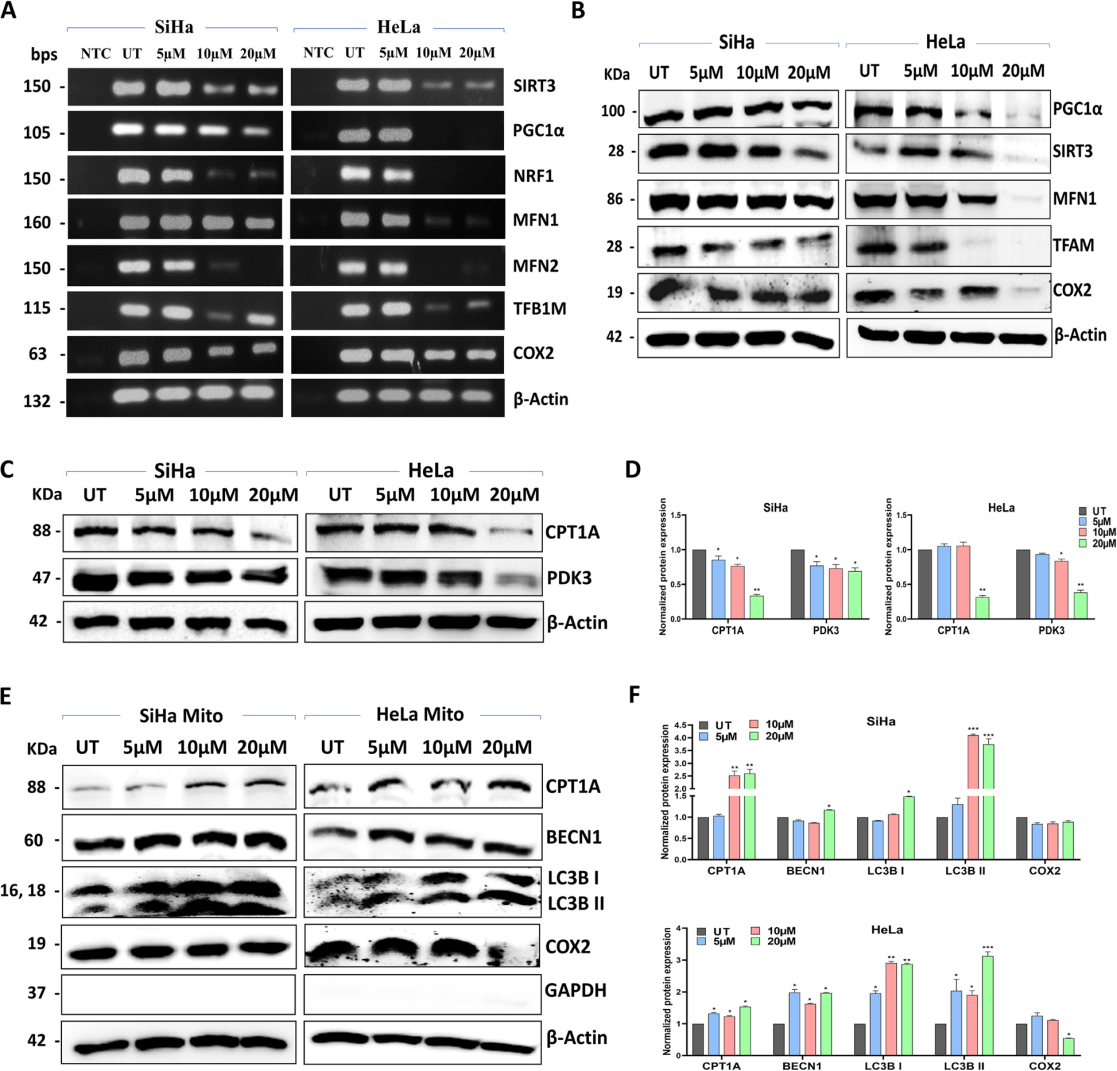
PC affects the expression of mitochondrial biogenesis genes and induces mitophagy in CC. **A** Representative agarose gel image of semiquantitative RT-PCR depicting the expression of *PGC1α, TFB1M, COX2, NRF1, MFN1, MFN2* and *SIRT3* in control and PC-exposed SiHa and HeLa cells. *ACTB* was used as a loading control (NC – Negative control). **B** Representative Western blot images showing the expression of PGC1α, TFAM, COX2, MFN1, and SIRT3 in PC-treated SiHa and HeLa cells. **C** and **D** Representative Western blot images and bar graphs of CPT1 A and PDK3 expression in control and PC-exposed SiHa and HeLa cells. **E** and **F** Representative Western blot images and bar graph showing the expression of CPT1 A, COX2, BECN1, LC3B I, and LC3B II in mitochondria isolated from control and PC-exposed SiHa and HeLa cells, respectively. *P < 0.05, **P < 0.01, and ***P < 0.001 indicates statistical significance

The mRNA levels of *SIRT3, PGC1 A,* *TFB1M, COX2,* *NRF1, MFN1,* and *MFN2* were examined because these genes and their corresponding proteins play crucial roles in mitochondrial function and biogenesis. The mRNA levels of these genes was significantly reduced at 20 µM in SiHa (Fig. 6A). At 10 µM and 20 µM PC treatment completely diminished the expression of mitochondrial biogenesis genes in HeLa (Supplementary Fig. 7 A). Treatment with 20 µM of PC reduced the protein expression of MFN1, PGC1α, SIRT3, TFAM, and COX2 in SiHa and HeLa cells (Fig. 6B). HeLa cells treated with 10 µM of PC decreased the expression of MFN1, PGC1α, TFAM, and COX2. A similar trend was observed for SIRT3, TFAM, and COX2 in SiHa cells exposed to 10 µM of PC. TFAM and COX2 levels were commonly downmodulated in SiHa and HeLa cells upon 5 µM of PC treatment (Supplementary Fig. 7B). In SiHa cells, the PDK3 level was downregulated at all three doses, however, in HeLa cells, substantial inhibition was noticed at 10 µM and 20 µM of PC (Fig. 6C and D). We observed an appreciable increase in the level of CPT1 A, LC3B I, LC3B II, and BECN1 in the mitochondrial fractions in response to PC treatment in both the cell lines (Fig. 6E and F).

**Fig. 7:**
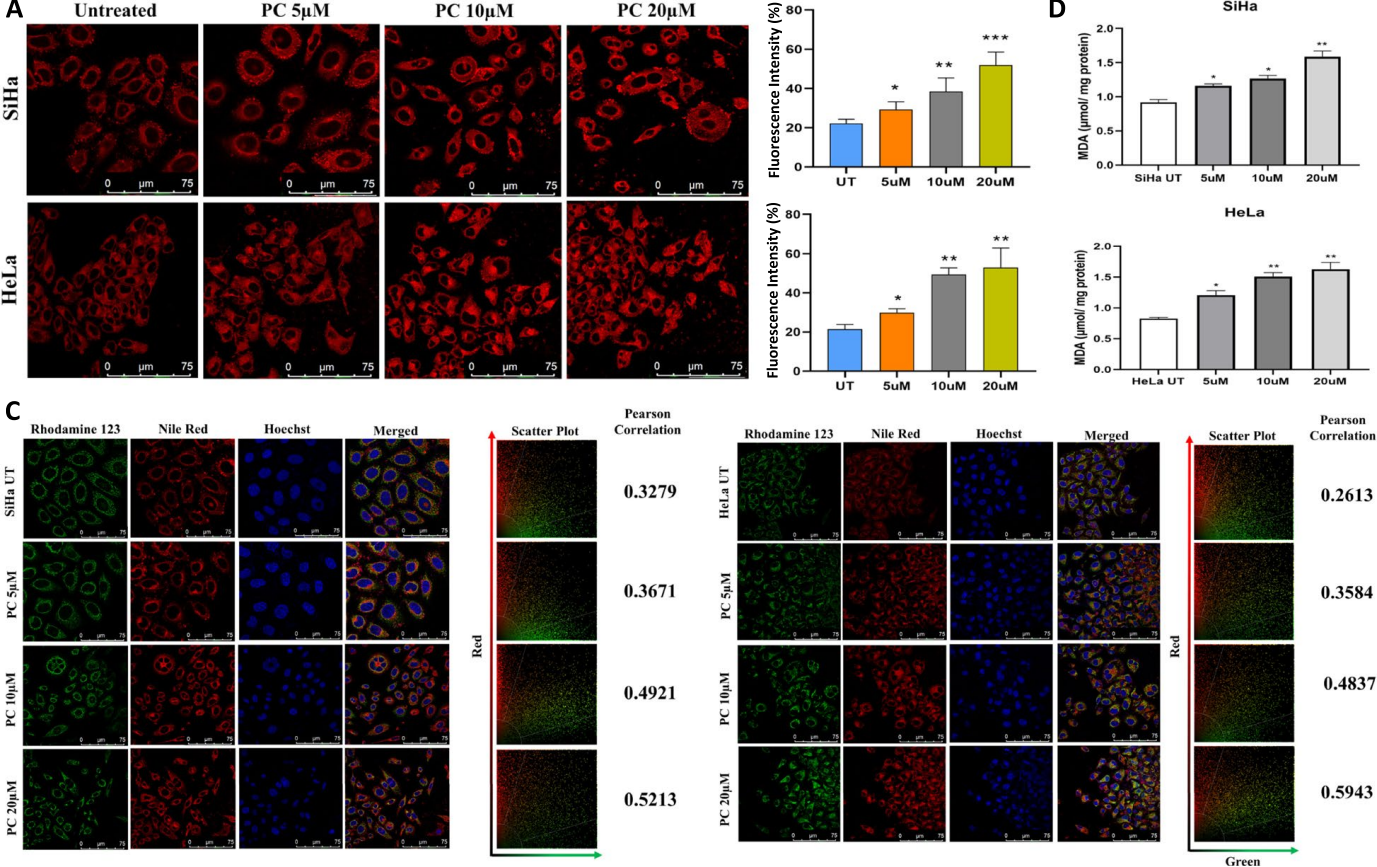
PC enhances lipid droplet accumulation and LPO in CC cells. **A** and **B** Representative confocal images and bar graph of Nile Red stained control and PC-exposed SiHa and HeLa cells, respectively. **C** Confocal images of control and PC-exposed SiHa and HeLa cells stained with Rhodamine-123 and Nile red to identify the mitochondrial localization of lipid droplets. **D** The bar graph represents the lipid peroxidation rate in control and PC-exposed SiHa and HeLa cells. The lipid peroxidation rate was estimated by normalized against the total protein. *P < 0.05, **P < 0.01, and ***P < 0.001 indicates statistical significance

PC-treated SiHa and HeLa cells showed more lipid droplet accumulation than untreated control cells. Nile red staining showed PC exposure to promote lipid droplet accumulation (Fig. 7A and B). Further, co-staining with Nile red and Rhodamine-123 followed by confocal microscopy suggested PC exposure to promote lipid accumulation in mitochondria. Pearson correlation coefficient was 0.32 for control SiHa cells that increased to 0.36, 0.49, and 0.52 upon treatment with 5 µM, 10 µM, and 20 µM of PC. Pearson correlation coefficient was 0.26 for control HeLa cells that increased to 0.35, 0.48, and 0.59 upon treatment with 5 µM, 10 µM, and 20 µM of PC (Fig. 7C). The malondialdehyde (MDA) levels increased with an increase in PC dose in a dose-dependent manner (Fig. 7D). Further, 20 µM of PC reduced the intensity of NAO, while 5 µM and 10 µM of PC treatment enhanced NAO intensity. These results suggested that PC can enhance lipid peroxidation in SiHa and HeLa cells.

### N-acetyl-l-cysteine (NAC) co-treatment decreases the lipotoxic effects of PC

**Fig. 8:**
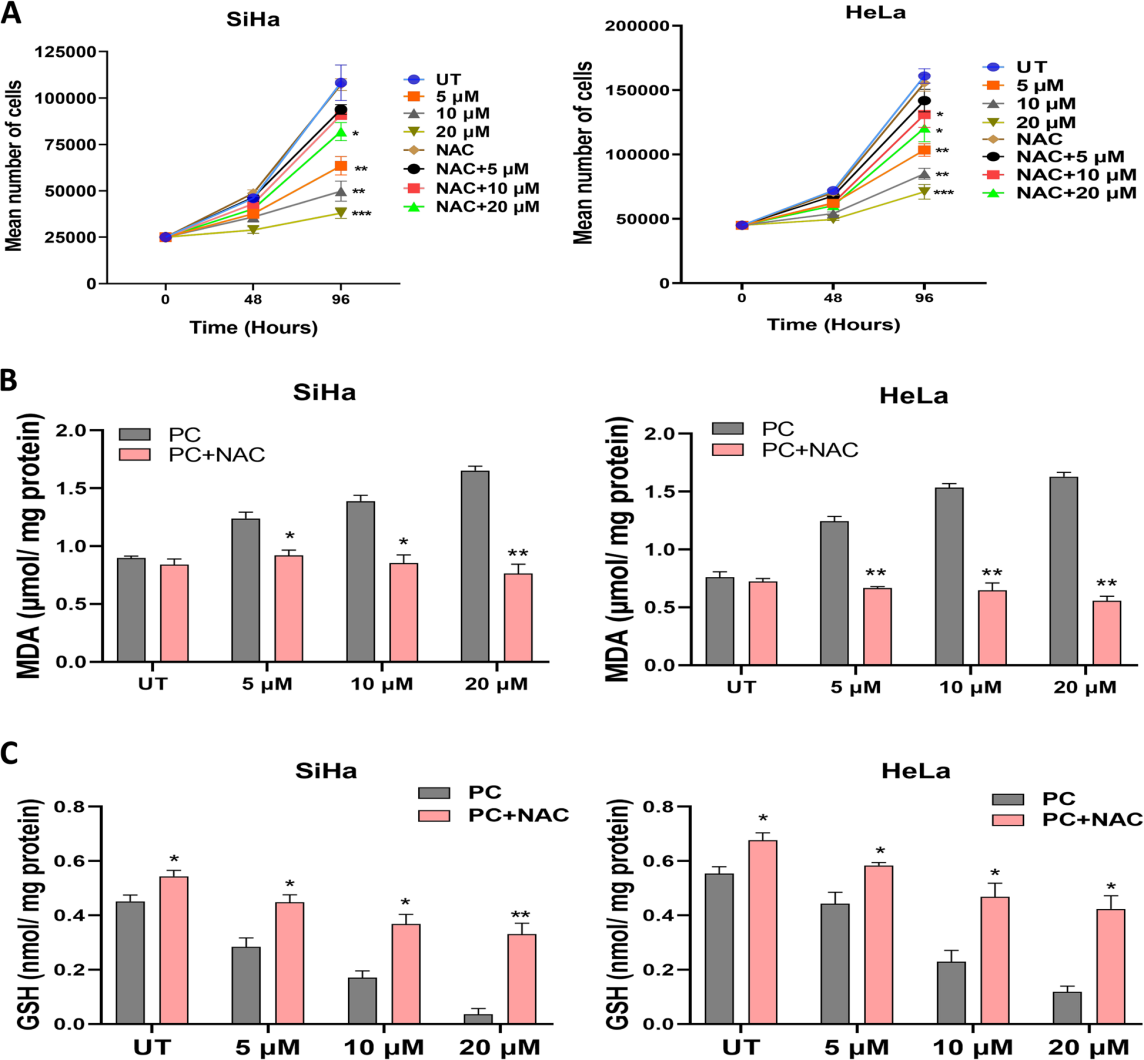
N-acetyl-l-cysteine co-treatment reverses the lipotoxic effects of PC. **A** Representative line graph showing the cell proliferation in control, PC-exposed, NAC-exposed, and combined treatment of PC and NAC in SiHa and HeLa cells. **B** The bar graph representing the lipid peroxidation rate upon co-incubation of NAC with PC in SiHa and HeLa cells. **C** The bar graph representing the GSH activity upon co-incubation of NAC with PC in SiHa and HeLa cells. *P < 0.05, **P < 0.01, and ***P < 0.001 indicates statistical significance

Co-exposure of NAC (750 µM) and PC significantly reduced the SiHa and HeLa cell proliferation than PC alone exposed cells (Fig. 8A). Co-incubation of NAC and PC significantly reversed the anti-proliferative effects of PC (Fig. 8A). The co-incubation of NAC and PC significantly reversed the PC-induced lipid peroxidation rate (Fig. 8B) with a concomitant increase in GSH enzymatic synthesis ability compared to the PC-only exposed cells (Fig. 8C). These results suggested that NAC treatment reverses the effect of PC on cell proliferation, lipid peroxidation, and GSH concentration.

### PC treatment increased the cytotoxic effects of PC

**Fig. 9:**
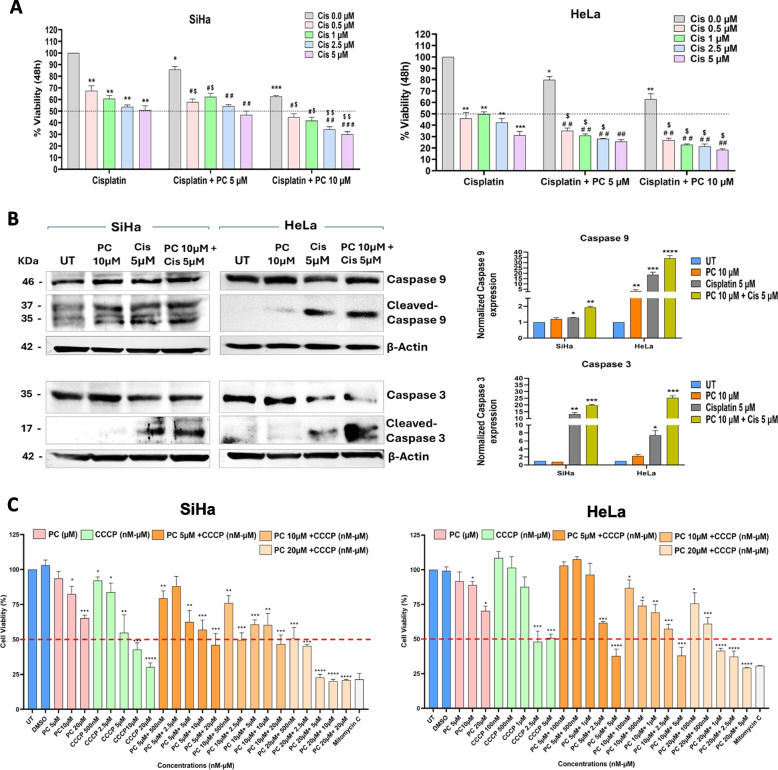
PC increases the cytotoxic effects of cisplatin. **A** Representative bar graph showing the percentage viability of SiHa and HeLa cells in response to cisplatin and co-exposure with cisplatin and PC (*Compared to untreated (Cis 0.0 μM); ^#^Compared to PC treatment; ^$^Compared to Cisplatin treatment). **B** Representative Western blot images and bar graph showing the expression of Caspase 9 and Caspase 3 in control, PC-exposed, Cisplatin-exposed, and combined treatment of PC and Cisplatin in SiHa and HeLa cells, respectively. **C** A representative bar graph shows the percentage viability of SiHa and HeLa cells in response to CCCP and co-exposure to CCCP and PC. Both PC and CCCP exposure induced cell death in SiHa and HeLa cells. However, the combination of CCCP with PC did not significantly increase cell death at the lower doses used in this study. *P < 0.05, **P < 0.01, and ***P < 0.001 indicates statistical significance

Cisplatin is the most common chemotherapeutic agent against CC. We used two doses of PC (5 µM, and 10 µM) and four doses of cisplatin (0.5 µM, 1 µM, 2.5 µM, and 5 µM) and their combination to assess the ability of PC to sensitize the cisplatin effects (Fig. 9A). A 48-h MTT assay revealed that a combination of PC and cisplatin showed more cytotoxicity than individual drugs in both the cell lines. In SiHa cells, 10 µM PC combined with varying doses of cisplatin showed 20% more cell death than individual cisplatin doses. Nevertheless, cisplatin’s cytotoxic effects were not significantly enhanced by the addition of 5 µM PC. In HeLa cells, 5 µM and 10 µM PC combined with various cisplatin concentrations greatly increased the cytotoxic effects compared to individual treatments (Fig. 9A). Further, we performed Western blot analysis to confirm PC and cisplatin-mediated apoptosis. Treatment with cisplatin alone and combination of PC with cisplatin resulted in an increase in cleaved caspase-9 levels in both SiHa and HeLa cells. Furthermore, cleaved caspase-3 levels were significantly elevated in both cell lines upon cisplatin alone and combination treatment of PC with cisplatin. (Fig. 9B). Our data shows that PC treatment can be used to improve the cytotoxic effects of cisplatin.

**Fig. 10:**
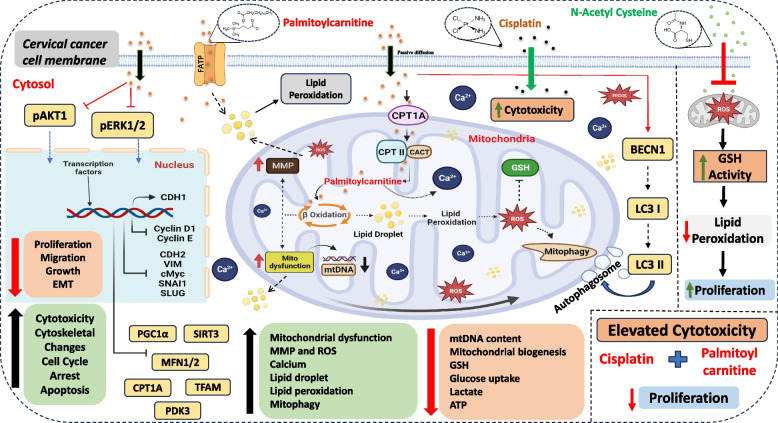
A schematic diagram representing the summary of the study. Palmitoyl carnitine (PC) treatment inhibits cervical cancer cell proliferation and migration by inducing oxidative stress, calcium overload, lipotoxicity, and mitochondrial dysfunction, while modulating key genes involved in apoptosis, autophagy, and EMT. The schematic illustrates the proposed mechanism and highlights the potential of PC, especially in combination with cisplatin, as a therapeutic strategy for cervical cancer

SiHa and HeLa cells were grouped into untreated, PC-treated, CCCP-treated, and PC + CCCP-treated to assess whether PC-mediated cytotoxicity is mitochondrial-dependent or independent. CCCP-induced cell death showed IC50 values of 5.44 µM for SiHa and 1.21 µM for HeLa cells. The combination treatment of CCCP with PC (5 µM and 10 µM) did not result in significant cytotoxicity in either cell line. Notably, a higher concentration of PC (20 µM) combined with CCCP (> 500 nM) showed significant cytotoxicity (less than 50% cell viability) compared to the untreated, PC-treated, and CCCP-treated groups (Fig. 9C). A schematic diagram representing the summary of the study is shown in Fig. 10.

## Discussion

Lipotoxicity and mitophagy can reduce tumour cell proliferation to suppress carcinogenesis [41, 42]. DOC2B is an epigenetically silenced gene that curbs metastasis by inhibiting EMT and senescence activation via increasing intracellular Ca^2+^ levels [6, 10]. DOC2B is a strong Wnt/beta-catenin pathway inhibitor and contributes to lipotoxicity and mitochondrial dysfunction in CC [11, 12]. Our recent data shows the presence of DOC2B in EVs collected from cells ectopically expressing DOC2B. Further, the transfer of these EVs to recipient SiHa cells showed a wide array of tumor-suppressive properties [13]. We showed that the re-expression of DOC2B can change the metabolome of EVs, especially the abundance of lipids, fatty acids, and steroids. PC is one of the metabolites whose abundance is significantly altered in response to the re-expression of DOC2B. Hence, we investigated the biological effect of PC in CC using cell lines and biochemical experiments. PC treatment reduced CC proliferation and growth via induction of mitochondrial dysfunction and lipotoxicity. PC treatment reduced the active form of AKT, ERK1/2, and members of EMT signaling pathways to suppress the proliferation, growth, and migration of CC cells. Notably, PC treatment increased the cytotoxic effects of cisplatin. Thus, reactivation of DOC2B or treatment with PC may be a novel therapeutic approach for CC management.

We first carried out a global metabolomic analysis to comprehend the effects of DOC2B overexpression on metabolite abundance in EVs. PCA analysis suggested that DOC2B overexpression significantly changed the global metabolite profile of EVs. DOC2B overexpression increased the abundance of glycerophospholipids, fatty acid esters, and steroids while decreasing the abundance of sphingolipids, amino acids, peptides, carbohydrates, and nucleosides. Biosynthesis of cardiolipin, phosphatidylethanolamine, phosphatidylcholine, thiamine metabolism, lactose degradation, De Novo Tri glycerol biosynthesis, pantothenate and CoA biosynthesis, and mitochondrial electron transport chain are the major pathways enriched upon DOC2B overexpression. However, we don’t know how DOC2B overexpression altered the metabolites abundance within EV and the biological effects of those changes.

PC, an ester derivative of carnitine, facilitates the transport of long-chain fatty acids into the inner membrane of the mitochondria for the process of fatty acid oxidation [20]. The intracellular transport of PC is mediated through passive diffusion or by Fatty Acid Transport Protein (FATP) family. PC can affect Na^+^/K^+^ and Ca^2+^ pumps, induce Ca2 + influx, pro‐inflammatory pathways, and have DHT-like effects [22]. In Type-II diabetes, PC measurement is proposed as a surrogate marker for lipotoxicity [43]. The present investigation is the first study to evaluate the biological effects of PC in CC. Hence, the present study investigated PC’s cytotoxic effects on SiHa and HeLa by MTT assay. Treatment with 30 µM of PC or above showed significant cytotoxicity in SiHa and HeLa cells. Our observation was consistent with previous studies that showed cytotoxic effects of PC on colon cancer (HT29 and HCT 116) [26], liver cancer (HepG2) [44], and prostate cancer (PC3) cells [22]. We showed that CC is more sensitive to PC than normal cells (HaCaT and fibroblast), oral cancer (Cal27), and breast cancer (MCF7). PC selectively targets CC cells due to their unique metabolic reprogramming and mitochondrial dysfunction. In contrast, normal cells possess more robust antioxidant defenses and greater metabolic flexibility, allowing them to adapt to metabolic stressors and maintain cellular homeostasis. Additionally, exposure to PC also inhibited the growth of CC cells. Our results indicate that PC treatment efficiently delays the growth and proliferation of CC cells.

CC cells exposed to PC showed substantial changes in cell morphology and induced apoptosis. The role of actin cytoskeletal network in proliferation, growth, migration, and apoptosis is well established [45, 46]. Further, actin derangement is also critical for the induction of apoptosis [47]. Hence, we investigated the PC effect on actin arrangement and cell morphology. Actin-phalloidin staining suggested changes in morphology and actin cytoskeleton arrangement upon PC treatment in CC cells. PC exposure reduced S-phase cells and induced cell death in CC cells. Thus, induction of apoptosis and S-phase arrest may be the reason for decreased cell proliferation and growth arrest following exposure to PC. Biologically, the reduced cell proliferation, growth, and migration of CC cells upon PC exposure could be connected to actin cytoskeleton rearrangement, S-phase arrest, and apoptosis.

The expression of critical proteins connected with cell proliferation, cell cycle, and survival has been examined to understand the molecular mechanism of PC-mediated anti-cancer activities. We found a negative correlation between PC treatment and expression of phosphorylated AKT1 and ERK1/2, Cyclin D1, Cyclin E, and cMYC. Further, downregulation of EMT pathway genes was also seen in CC cells exposed to PC. Upregulation of CDH2, SNAI1, SLUG, VIM, and cMYC alone can enhance cell survival, proliferation, migration, and therapy resistance in CC [10, 32, 48]. Hence, it is possible that the anti-cancer properties of PC were aided by a decrease in the active form AKT1 and ERK1/2 and the downregulation of Cyclin D1, Cyclin E, and EMT pathway genes.

Previous studies reported that PC can induce mitochondrial dysfunction [49, 50]. Accumulation of PC caused PC-induced insulin resistance in mice muscle cells [49]. The O^2−^ produced in response to PC exposure relates to the initiation of apoptosis in HT-29 cells [24]. Glutathione deletion and H_2_O_2_ production upon PC treatment may abrogate the survival of colorectal cancer cells [26]. PC treatment increased ROS production and decreased MMP in cardiomyocytes [51]. In HepG2 cells, PC treatment induced oxidative stress via elevating ROS [44]. Besides, there was dose dependent GSH enzyme reduction in both SiHa and HeLa cells in response to PC treatment. Our study findings revealed that PC treatment affected mitochondrial morphology, average branch length, branch diameter, perimeter, and circularity. Studies have consistently reported that cells initiated for apoptosis show an increase in MMP levels [52, 53]. We observed a dose-dependent increase in MMP upon PC exposure in both cell lines. Besides, cells with metabolic defects can show a simultaneous increase in ROS and MMP [54]. ROS and calcium measurement suggested mitochondrial and intracellular oxidative stress and calcium overload in PC-treated cells. This observation is consistent with our previous observation that DOC2B re-introduction increased ROS and calcium overload in SiHa cells [12]. In prostate cancer, treatment with high PC-induced calcium influx [22]. PC exposure showed a dose-dependent reduction in the uptake of glucose and production of lactate in both cell lines. Cancer cells show metabolic reprogramming, such as an increase in the uptake of glucose and the production of lactate to sustain the high energy demand [55]. The elevation in ATP levels at lower concentrations is due to the increased intake of PC and subsequent β-oxidation. However, PC accumulation leads to increased lipotoxicity and ROS production, which alters mitochondrial dynamics and results in a reduction in ATP levels. These data suggested that PC can induce metabolic reprogramming and mitochondrial dysfunction in CC.

During lipid peroxidation, ROS targets the lipids to produce malondialdehyde (MDA) and 4-hydroxy-2-nonenal (HNE) intermediate. Both MDA and HNE are toxic to cells and can activate autophagy and apoptosis [56, 57]. Within mitochondria, cardiolipin, ceramide, and sphingosine-1-phosphate operate as mitophagy signals [58–60]. Cardiolipin, present in the inner mitochondrial membrane, is particularly vulnerable to lipid peroxidation [61]. Mitochondria are considered the main site of HNE generation, and epoxide-containing cardiolipin can trigger autophagy [62]. Considering the crucial role of ROS in lipid peroxidation, we investigated the lipid peroxidation rate upon PC exposure. We found a dose-dependent increase in total and mitochondrial ROS and lipid peroxidation rate upon PC exposure in both cell lines. This suggests that PC exposure can trigger lipid peroxidation through several mechanisms. One primary mechanism involves the disruption of mitochondrial function, where PC integrates into the mitochondrial membrane, leading to an imbalance in fatty acid metabolism. This disruption results in the accumulation of ROS within the mitochondria. Elevated ROS levels initiate lipid peroxidation, targeting polyunsaturated fatty acids in cell membranes and generating lipid radicals, which further propagate oxidative damage. Furthermore, PC role in enhancing fatty acid uptake into mitochondria exacerbates oxidative stress by overwhelming cellular antioxidant defenses, leading to increased lipid peroxidation. This oxidative stress and subsequent lipid peroxidation contribute to mitochondrial dysfunction and activate cell death pathways such as apoptosis, particularly in cancer cells with compromised antioxidant systems.

To understand whether the lipid peroxidation is ROS-dependent or independent, we chelated the cells with 750 µM NAC and performed a lipid peroxidation assay. Treatment with an antioxidant agent reduced the lipid peroxidation rate, suggesting that PC-induced lipid peroxidation is a ROS-dependent process. Lipid droplets and mitochondria are closely linked and can promote mitophagy [63]. PC treatment increases cellular and mitochondrial lipid droplet accumulation in a dose-dependent manner and may promote mitophagy. We show that PC can induce lipid peroxidation and lipid droplet accumulation in CC cells.

Considering the biological effect of lipid peroxidation on autophagy, it is possible that PC-induced ROS-triggered lipid peroxidation may have promoted mitophagy. Reduced glutathione (GSH) is recognized to be essential for preventing lipid peroxidation by ROS [56]. Glutathione is known to reduce oxidative stress-induced mitochondrial damage. Reduced GSH levels have been shown to increase oxidative stress and reduce mitochondrial function [64]. Herein, we observed PC to promote lipid peroxidation and lipid droplet accumulation in CC cells. The reduction in the GSH level observed may have contributed to an increase in ROS and lipid peroxidation in PC-treated cells. Cells undergoing apoptosis show peroxidation of cardiolipins, which is required to release cytochrome-c [65]. Hence, we measured the cardiolipin peroxidation in response to PC treatment by NAO staining. Treatment with 20 µM of PC significantly decreased NAO intensity, suggesting cardiolipin peroxidation. Thus, we propose that PC treatment-induced ROS and simultaneous loss of GSH may have contributed to enhanced lipid peroxidation, lipid droplet accumulation, and cardiolipin peroxidation to promote mitophagy in CC. Carnitine-palmitoyl CoA transferase 1 (CPT1 A) is an enzyme crucial for long-chain fatty acid uptake inside the mitochondria for beta-oxidation [66].

In our study, western blotting of mitochondrial lysate suggested that PC treatment increased the mitochondrial CPT1 A. Interestingly, the intracellular CPT1 A level was significantly reduced in response to PC exposure. The role of CPT1 A in inducing oxidative stress is already reported [67, 68]. Thus, an increase in CPT1 A level within mitochondria upon PC exposure correlated with an increase in ROS, calcium, lipid peroxidation, lipid droplet accumulation, and mitophagy. Pyruvate dehydrogenase kinase 3 (PDK3) is a mitochondrial enzyme critical for the conversion of pyruvate to acetyl-CoA and CO_2_ during glucose metabolism [69]. PC exposure decreased PDK3 levels in a dose-dependent manner. Nonetheless, additional, comprehensive mechanistic investigations are required to comprehend the importance of changes in CPT1 A and PDK3 expression with PC treatment.

Beclin-1, LC3B I, and LC3B II are inducers of autophagy [70]. A rise in Beclin-1, LC3B I, and LC3B II levels within mitochondria is suggestive of induction of mitophagy following PC treatment. Mitophagy and mitochondrial biogenesis are intimately connected [71]. Our findings show that PC treatment significantly reduced the expression of mitochondrial biogenesis-related proteins such as MFN1, PGC1α, SIRT3, TFAM, and COX2. Hence, we propose that an increase in ROS and reduced antioxidant level might have contributed to excessive ROS production, lipid peroxidation, mitophagy, and mito-lipotoxicity. Our study showed the crucial role of mitochondria in PC-induced cytotoxicity.

Cisplatin is the most used therapeutic drug for CC treatment [72]. Reduced accumulation of intracellular cisplatin is one of the mechanisms for cisplatin resistance. Besides, hyperactivation of PI3 K/AKT, cMYC, and ERK1/2 have shown to contribute to cisplatin resistance [5, 73]. Several studies used combination therapies to overcome cisplatin resistance [74, 75]. Further, combining cisplatin with natural compounds has been shown to overcome cisplatin resistance and increase the cytotoxic effect of cisplatin by lowering the IC50 value [76]. Thus, including PC during cisplatin treatment could significantly enhance its cytotoxic effects and apoptosis. This is the first study to demonstrate that PC enhances the cytotoxic and pro-apoptotic effects of cisplatin in CC cells. This is particularly important to enhance the treatment effects and to reduce the dose-responsive side effects. Interestingly, among the cell lines tested, CC cells were more sensitive to PC than other cells. The combined treatment of PC with cisplatin was less sensitive in normal, oral, and breast cancer cells. A previous colorectal cancer study showed that PC exposure induced more cytotoxicity in colon cancer lines, while MCF7 was less sensitive to PC. These differences in sensitivity to PC were partially due to the inability of colorectal cancer cells to prevent oxidative stress via glutathione-redox coupling [26]. Our data suggested that PC can be used along with cisplatin to increase the cytotoxicity and therapeutic outcome.

While this study provides valuable insights into PC-induced metabolic reprogramming in CC cells, certain limitations should be acknowledged. The findings of the study is primarily based on cervical cancer cell lines which may not fully recapitulate the complexity of the tumor microenvironment. In-vivo validation using animal models may be undertaken to evaluate the efficacy, pharmacokinetics, biodistribution, and potential side effects of PC. While we showed that PC induced lipotoxicity and mitochondrial dysfunction may have contributed to decreased survival of CC cells, the precise molecular mechanisms underlying PC-induced metabolic alterations and their relevance across different cancer types remain to be fully elucidated. The long-term effects of PC treatment and its impact on resistance mechanisms may be investigated, including potential resistance mechanisms, were not assessed. Clinical trials to assess PC’s safety and efficacy in CC patients, particularly in combination with cisplatin may be explored as part of future studies.

In summary, our findings demonstrated that DOC2B can impact the composition of EVs, notably the abundance of PC. The present study established the anticancer properties of PC and the associated mechanism in CC. PC treatment negatively impacted CC cell growth, proliferation, and migration by inducing cell cycle arrest and apoptosis. PC treatment induced oxidative stress, calcium overload, and lipotoxicity. Mechanistically, PC affects the proliferation, survival, cell cycle progression, autophagy, and EMT genes. PC-exposed CC shows oxidative stress, calcium overload, mitochondrial dysfunction, lipotoxicity, lipid droplet accumulation, and mitophagy. PC elevated intracellular ROS to inhibit cell proliferation. Thus, PC, in combination with cisplatin, can be an excellent therapeutic agent for CC. Our findings may be extremely useful in developing new intervention techniques and molecular-focused therapy for CC.

## Supplementary Information

Supplementary Material 1: Supplementary Table 1 List of RT-PCR Primers

Supplementary Material 2: Supplementary Fig. 1 Size distribution of DOC2B EVs and effect of Palmitoylcarnitine on cell proliferation A) Size and intensity distribution of EVs using Zetasizer Nano-ZS instrument. B) Cytotoxic effects of PC on various cell lines. The bar graph represents the percentage cell viability of SiHa, HeLa, Fibroblast, HaCaT, Cal27, and MCF7 cells in response to PC exposure for 48 h as analyzed by MTT assay. C) The bar graph represents the percentage cell viability of SiHa, and HeLa cells in response to PC exposure for 24 h as analyzed by MTT assay. Data presented are mean ± SD of three independent experiments in triplicate)

Supplementary Material 3: Supplementary Fig. 2 DOC2B EVs enriched Glycerophospholipid pathway: Metabolite enrichment in DOC2B EVs compared to control EVs was plotted by PATHVIEW. Red represents up-regulated metabolites, green represents down-regulated metabolites and violet represents no significant difference between control EVs and DOC2B EVs

Supplementary Material 4: Supplementary Fig. 3 Pathway enrichment analysis using MetaboAnalyst. The metabolic set enrichment analysisof metabolomics data generated from EVs harvested from DOC2B-SiHa and Vector-SiHa

Supplementary Material 5: Supplementary Fig. 4 AO/EtBr staining and cell cycle flow-cytometry histograms. A) The bar graph showing the percentage of viable and apoptotic cells in AO/EtBr stained SiHa and HeLa cells upon PC exposure. B) Representative flow cytometry histograms showing cell cycle distribution in control and PC-treated SiHa and HeLa cells. The histograms illustrate the percentage of cells in apoptotic, G0/G1, S, and G2/M phases, highlighting PC-induced cell cycle arrest. *P < 0.05, **P < 0.01, and ***P < 0.001 indicates statistical significance

Supplementary Material 6: Supplementary Fig. 5 Quantification of mitochondrial morphology. The bar graph represents the quantification of mitochondrial morphology in control and PC-treated SiHa and HeLa cells. *P < 0.05, **P < 0.01, and ***P < 0.001 indicates statistical significance

Supplementary Material 7: Supplementary Fig. 6 Mitochondrial Mass, Intracellular lactate, and ATP A) The bar graph represents PC-treated SiHa and HeLa cells stained with NAO. B) The bar graph represents intracellular lactate levels in control and PC-exposed SiHa and HeLa cells. C) The bar graph represents intracellular ATP levels in control and PC-exposed SiHa and HeLa cells. The biochemical assay data were normalized using total protein. *P < 0.05, **P < 0.01, and ***P < 0.001 indicates statistical significance

Supplementary Material 8: Supplementary Fig. 7 Quantification of mitochondrial biogenesis genes at mRNA and Protein level.The bar graph represents the quantification of gene expression at mRNA level in control and PC-treated SiHa and HeLa cells.The bar graph represents the densitometric quantification of gene expression at protein level in control and PC-treated SiHa and HeLa cells *P < 0.05, **P < 0.01, and ***P < 0.001 indicates statistical significance

## Data Availability

No datasets were generated or analysed during the current study.

## Abbreviations

ACTB: Actin Beta
ANOVA: Analysis of Variance
AO: Acridine Orange
ATCC: American Type Culture Collection
ATP: Adenosine Triphosphate
BECN1: Beclin-1
BSA: Bovine Serum Albumin
CC: Cervical Cancer
CCCP: Carbonyl cyanide m-chlorophenylhydrazone
CCND1: Cyclin D1
CCNE: Cyclin E
CDH1: E-Cadherin-1
COA: Coenzyme A
COX2: Cyclooxygenase 2
CPT1 A: Carnitine Palmitoyltransferase 1A
DCFDA: Dichlorofluorescin Diacetate
DHT: Dihydrotestosterone
DMEM: Dulbecco’s Modified Eagle Medium
DMSO: Dimethyl Sulfoxide
DNA: Deoxyribonucleic Acid
DOC2B: Double C-2-Like Domain Beta
EDTA: Ethylenediaminetetraacetic Acid
EGTA: Ethylene Glycol Tetraacetic Acid
EMT: Epithelial To Mesenchymal Transition
ERK1/2: Extracellular Signal-Regulated Kinase 1/2
EtBr: Ethidium Bromide
EVs: Extracellular Vesicles
GAPDH: Glyceraldehyde 3-Phosphate Dehydrogenase
GFP: Green fluorescent protein
GSH: Glutathione
HBSS: Hanks’ Balanced Salt Solution
HCOOH: Formic Acid
HMDB: Human Metabolome Database
HNE: 4-Hydroxy-2-Nonenal
HPLC: High-Performance Liquid Chromatography
HPV: Human Papillomavirus
HRP: Horse Radish Peroxidase
KEGG: Kyoto Encyclopedia of Genes And Genomes
LC3B: Microtubule-associated protein 1A/1B-light chain 3
MCF7: Michigan Cancer Foundation-7
MDA: Malondialdehyde
MFN1 and 2: Mitochondrial Fusion Protein 1 and 2
MID: Munc Interacting Domain
MMP: Mitochondrial Membrane Potential
MTT: 3-(4,5-Dimethylthiazol-2-Yl)-2,5-Diphenyltetrazolium Bromide
NAC: N-Acetylcysteine
NAO: Nonyl Acridine Orange
NRF1: Nuclear Respiratory Factor 1
NS: NanoSight
NTA: Nanoparticle Tracking Analysis
PBS: Phosphate Buffered Saline
PC: Palmitoyl Carnitine
PCA: Principal Components Analysis
PCR: Polymerase Chain Reaction
PDK3: Pyruvate Dehydrogenase Kinase 3
PGC1α: Peroxisome Proliferator-Activated Receptor-Gamma Coactivator
PI: Propidium iodide
PI3 K-AKT: Phosphatidylinositol 3-Kinase (Pi3K) And AKT/Protein Kinase B
PIPES: Piperazine-N,N′-Bis (2-Ethanesulfonic Acid)
RNA: Ribonucleic Acid
ROS: Reactive Oxygen Species
RT-PCR: Reverse Transcription polymerase chain reaction
SDS-PAGE: Sodium Dodecyl Sulfate–Polyacrylamide Gel Electrophoresis
SIRT3: Sirtuin-3
SLUG: Zinc Finger Protein
SNAI1: Snail Family Transcriptional Repressor 1
STR: Short Tandem Repeats
TBST: Tris-Buffered Saline + Tween 20
TCA: Tricarboxylic Acid Cycle
TFAM: Mitochondrial Transcription Factor A
TOF–MS: Time-Of-Flight Mass Spectrometry
TRITC: Tetramethylrhodamine
VIM: Vimentin
