# Supplementary material for: The ester derivative Palmitoylcarnitine abrogates cervical cancer cell survival by enhancing lipotoxicity and mitochondrial dysfunction
Source: Cell Commun Signal. 2025 May 3;23:213. doi: 10.1186/s12964-025-02218-8 (PMC12048960; doi:10.1186/s12964-025-02218-8)
Supplement: Supplementary file 1 — Supplementary Material 1: Supplementary Table 1 List of RT-PCR Primers [file 12964_2025_2218_MOESM1_ESM.docx]

**Supplementary Table 1:** List of RT-PCR Primers

| **Primer Name** | **Primer Sequence (5'-3')** | **Annealing Temperature** | **Product Size** |
| --- | --- | --- | --- |
| PGC1A-F | TTTCTGGGTGGACTCAAG | 59°C | 105bp |
| PGC1A-R | GTCTAGTGTCTCTGTGAG |  |  |
| TFB1M-F | ATGGCTCAGTACCTCTGCAATG | 56°C | 115bp |
| TFB1M-R | TGGGCTGTATCAAGGGAGTGA |  |  |
| MT-COX2-F | CCGACTACGGCGGACTAATC | 57°C | 63bp |
| MT-COX2-R | CGCCTGGTTCTAGGAATAATGG |  |  |
| SIRT3-F | ACCCAGTGGCATTCCAGAC | 59°C | 122bp |
| SIRT3-R | GCTTGGGGTTGTGAAAGAAGAA |  |  |
| NRF1-F | GCAAATGTCCGGAGTGATGT | 58°C | 120bp |
| NRF1-R | ATACAAAAGGTCTTCCCGCC |  |  |
| MFN1-F | TGTTTTGGTCGCAAACTCTG | 55°C | 160bp |
| MFN1-R | CTGTCTGCGTACGTCTTCCA |  |  |
| MFN2-F | ATTCAGAAAGCCCAGGGCATG | 55°C | 150bp |
| MFN2-R | GACCGTGTGCTGCTCAAACTTG |  |  |
| ACTB-F | GACGACATGGAGAAAATCTG | 60°C | 132bp |
| ACTB-R | ATGATCTGGGTCATCTTCTC |  |  |
