# Supplementary figures and images for: The ester derivative Palmitoylcarnitine abrogates cervical cancer cell survival by enhancing lipotoxicity and mitochondrial dysfunction
Source: Cell Commun Signal. 2025 May 3;23:213. doi: 10.1186/s12964-025-02218-8 (PMC12048960; doi:10.1186/s12964-025-02218-8)

## Slide 1
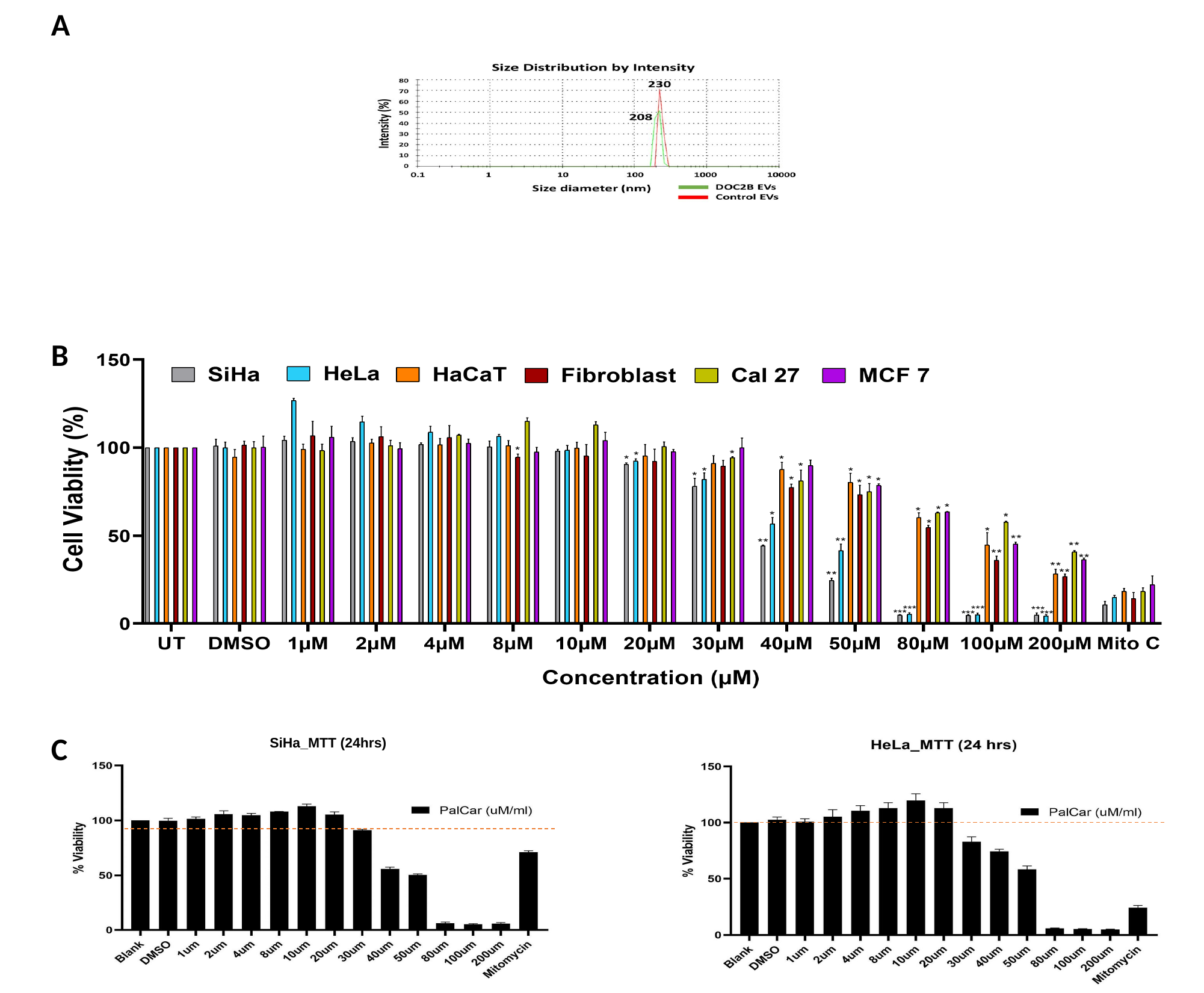

A
B
C
SiHa_MTT (24hrs)

Supplement: Supplementary file 2 — Supplementary Material 2: Supplementary Fig. 1 Size distribution of DOC2B EVs and effect of Palmitoylcarnitine on cell proliferation A) Size and intensity distribution of EVs using Zetasizer Nano-ZS instrument. B) Cytotoxic effects of PC on various cell lines. The bar graph represents the percentage cell viability of SiHa, HeLa, Fibroblast, HaCaT, Cal27, and MCF7 cells in response to PC exposure for 48 h as analyzed by MTT assay. C) The bar graph represents the percentage cell viability of SiHa, and HeLa cells in response to PC exposure for 24 h as analyzed by MTT assay. Data presented are mean ± SD of three independent experiments in triplicate) [file 12964_2025_2218_MOESM2_ESM.pptx]

## Slide 1
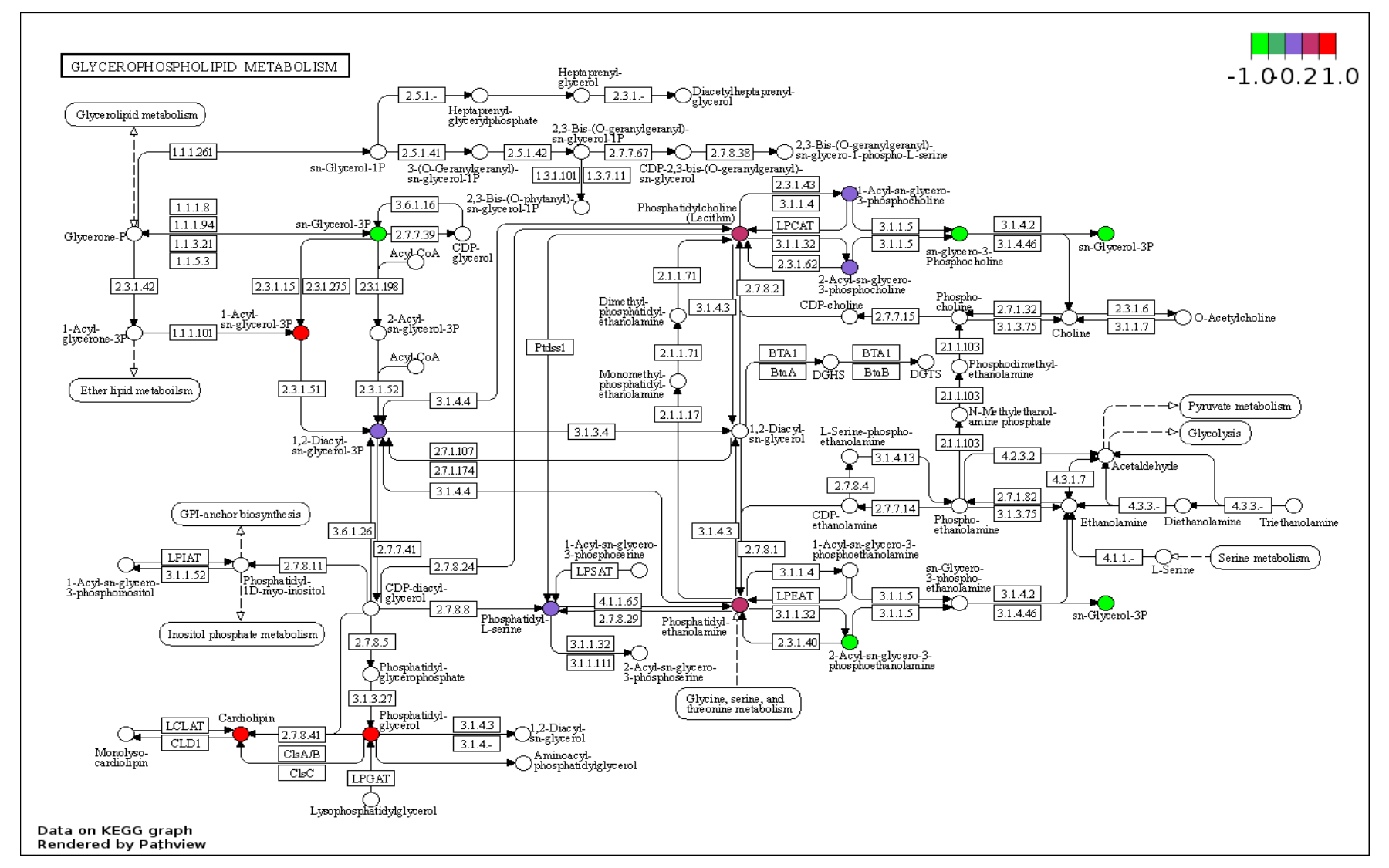

Supplement: Supplementary file 3 — Supplementary Material 3: Supplementary Fig. 2 DOC2B EVs enriched Glycerophospholipid pathway: Metabolite enrichment in DOC2B EVs compared to control EVs was plotted by PATHVIEW. Red represents up-regulated metabolites, green represents down-regulated metabolites and violet represents no significant difference between control EVs and DOC2B EVs [file 12964_2025_2218_MOESM3_ESM.pptx]

## Slide 1
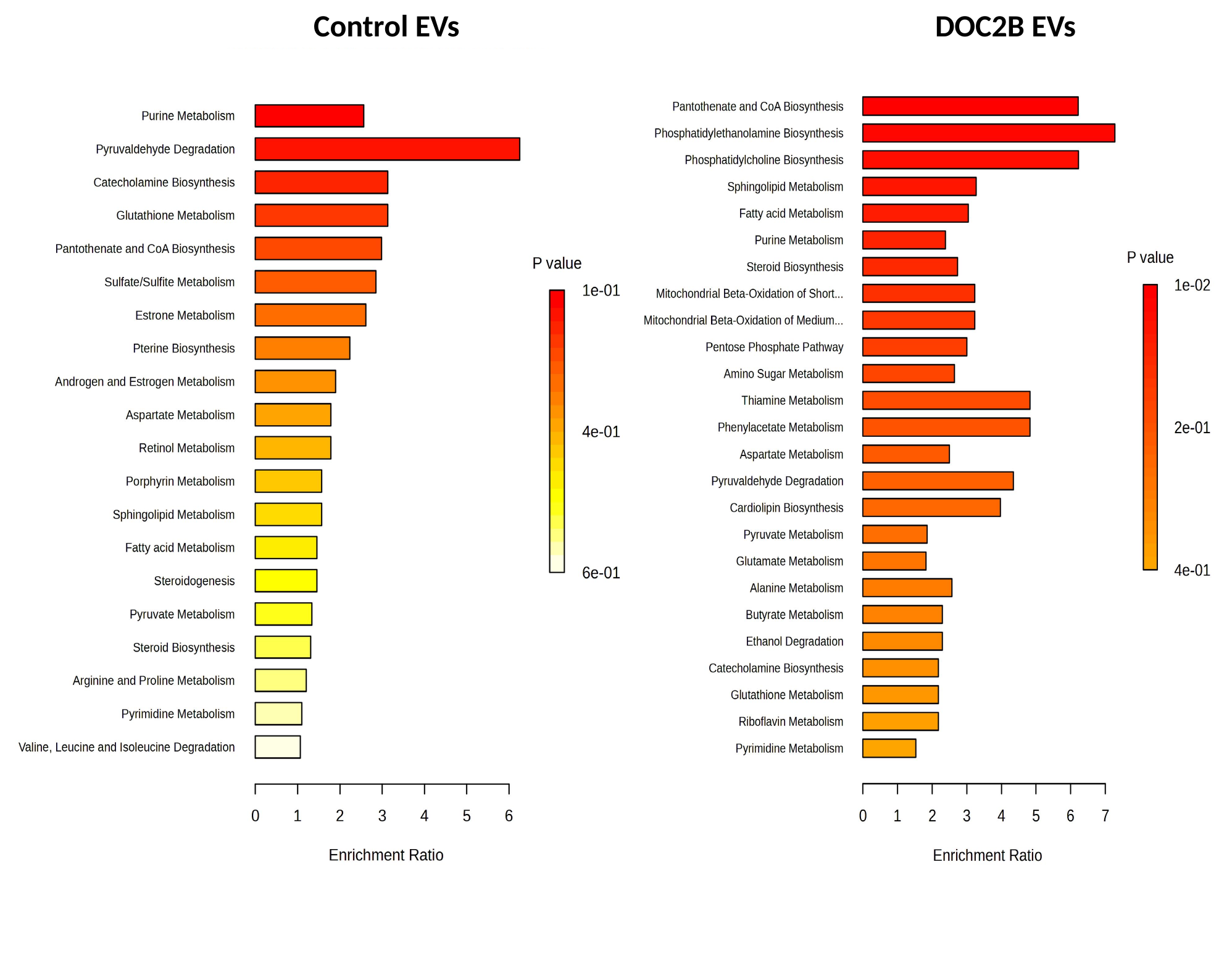

Control EVs
DOC2B EVs

Supplement: Supplementary file 4 — Supplementary Material 4: Supplementary Fig. 3 Pathway enrichment analysis using MetaboAnalyst. The metabolic set enrichment analysisof metabolomics data generated from EVs harvested from DOC2B-SiHa and Vector-SiHa [file 12964_2025_2218_MOESM4_ESM.pptx]

## Slide 1
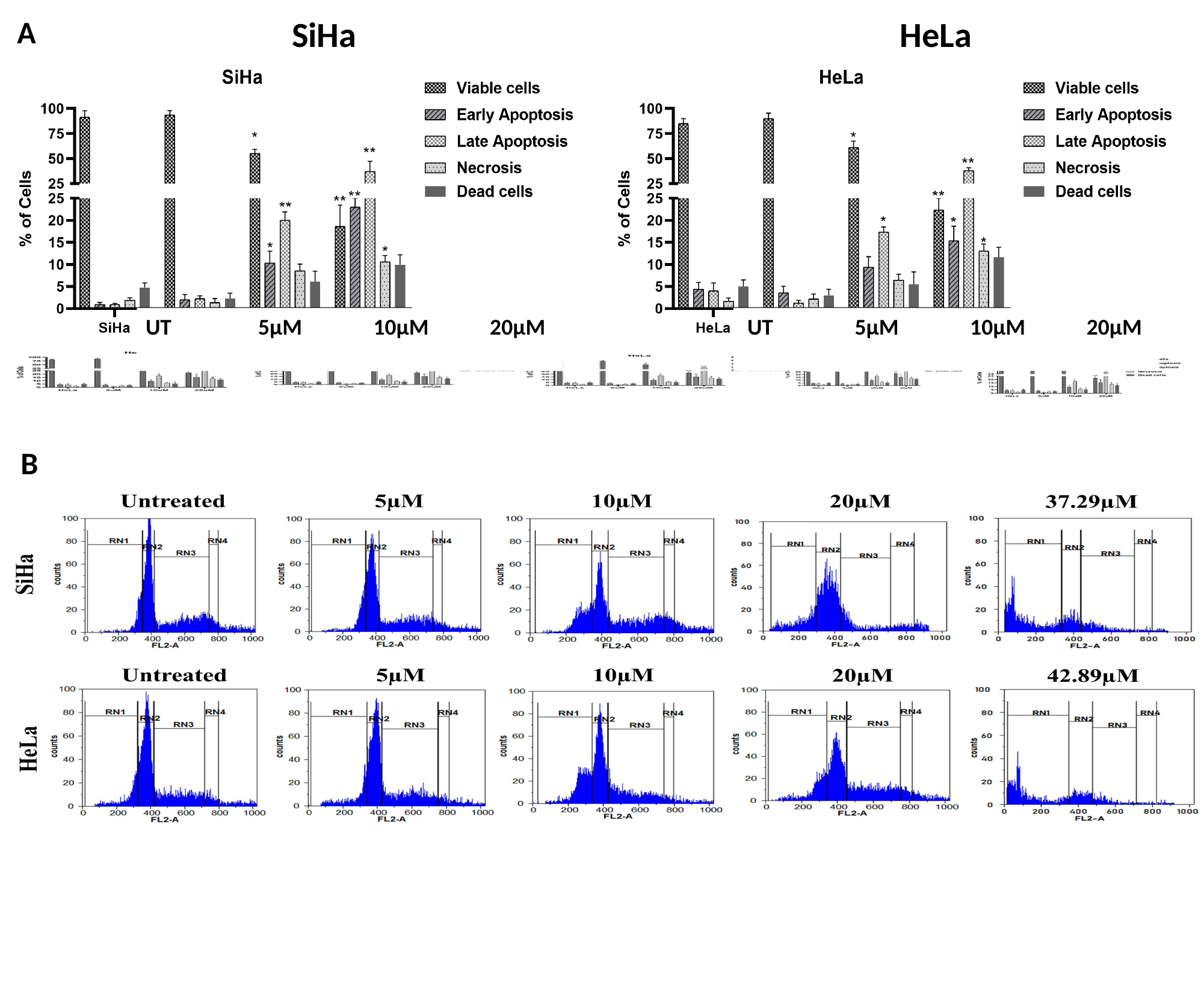

A
SiHa
HeLa
UT 5µM 10µM 20µM
 UT 5µM 10µM 20µM
B

Supplement: Supplementary file 5 — Supplementary Material 5: Supplementary Fig. 4 AO/EtBr staining and cell cycle flow-cytometry histograms. A) The bar graph showing the percentage of viable and apoptotic cells in AO/EtBr stained SiHa and HeLa cells upon PC exposure. B) Representative flow cytometry histograms showing cell cycle distribution in control and PC-treated SiHa and HeLa cells. The histograms illustrate the percentage of cells in apoptotic, G0/G1, S, and G2/M phases, highlighting PC-induced cell cycle arrest. *P < 0.05, **P < 0.01, and ***P < 0.001 indicates statistical significance [file 12964_2025_2218_MOESM5_ESM.pptx]

## Slide 1
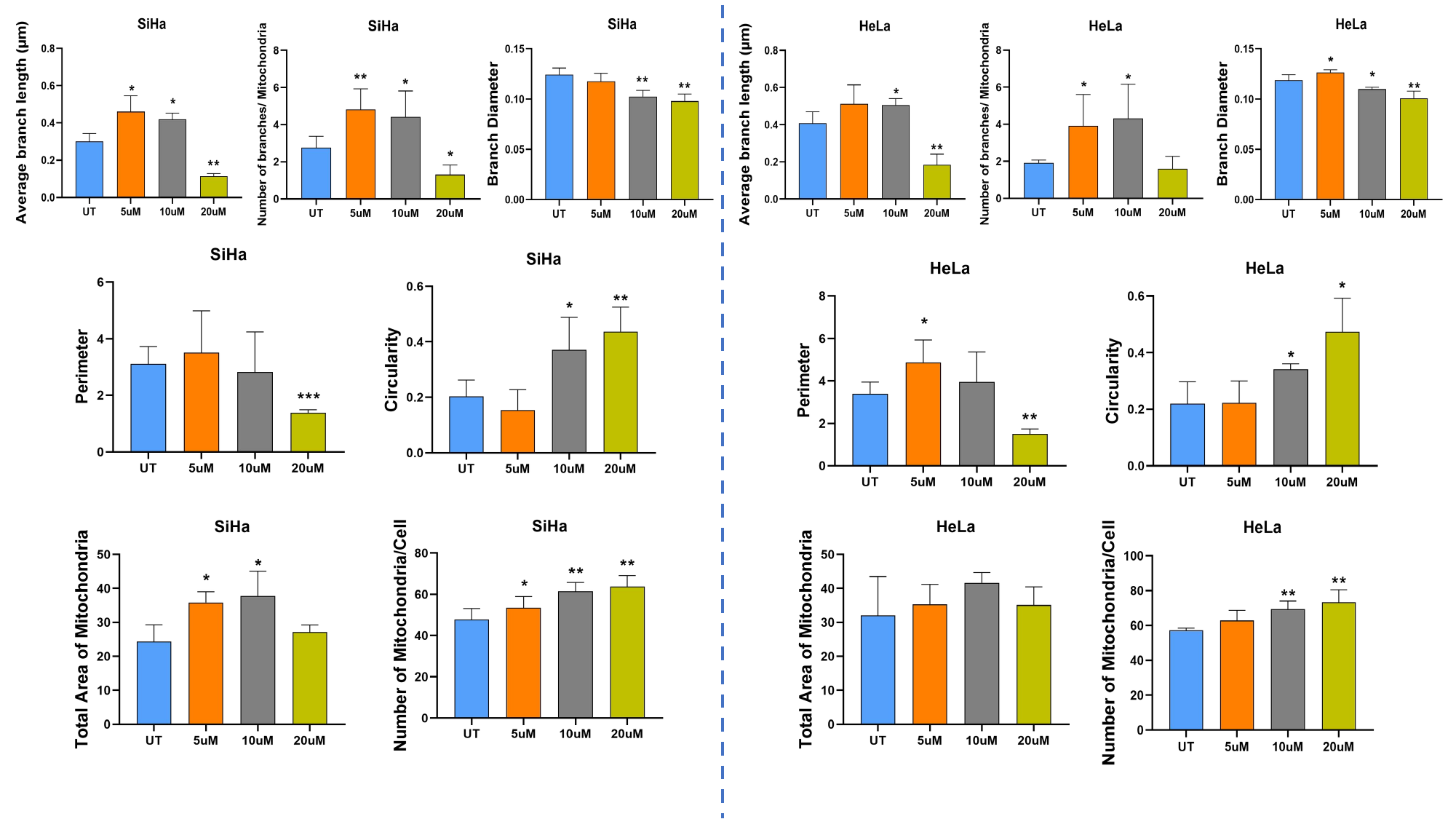

Supplement: Supplementary file 6 — Supplementary Material 6: Supplementary Fig. 5 Quantification of mitochondrial morphology. The bar graph represents the quantification of mitochondrial morphology in control and PC-treated SiHa and HeLa cells. *P < 0.05, **P < 0.01, and ***P < 0.001 indicates statistical significance [file 12964_2025_2218_MOESM6_ESM.pptx]

## Slide 1
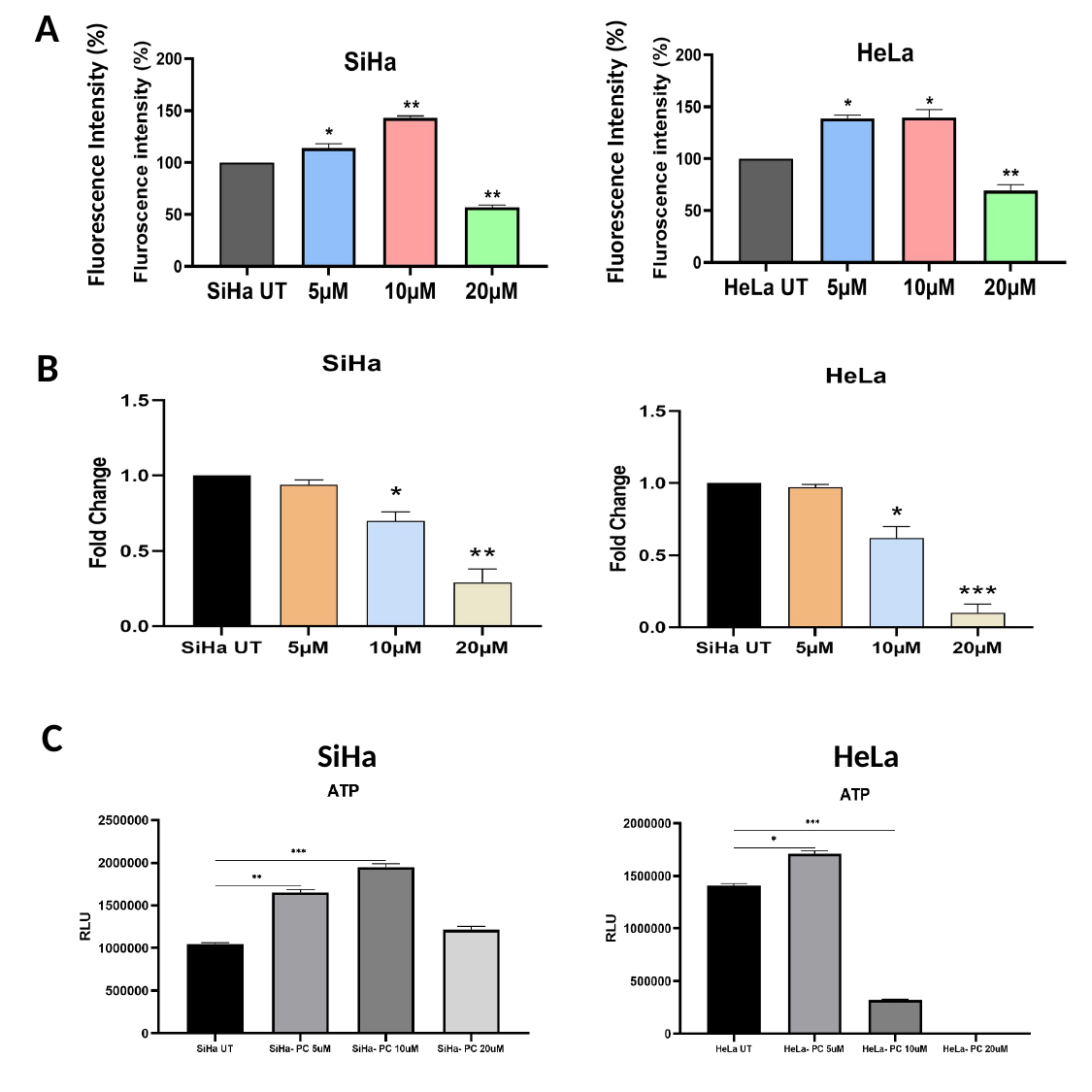

A
Fluorescence Intensity (%)
Fluorescence Intensity (%)
B
C
SiHa
HeLa

Supplement: Supplementary file 7 — Supplementary Material 7: Supplementary Fig. 6 Mitochondrial Mass, Intracellular lactate, and ATP A) The bar graph represents PC-treated SiHa and HeLa cells stained with NAO. B) The bar graph represents intracellular lactate levels in control and PC-exposed SiHa and HeLa cells. C) The bar graph represents intracellular ATP levels in control and PC-exposed SiHa and HeLa cells. The biochemical assay data were normalized using total protein. *P < 0.05, **P < 0.01, and ***P < 0.001 indicates statistical significance [file 12964_2025_2218_MOESM7_ESM.pptx]

## Slide 1
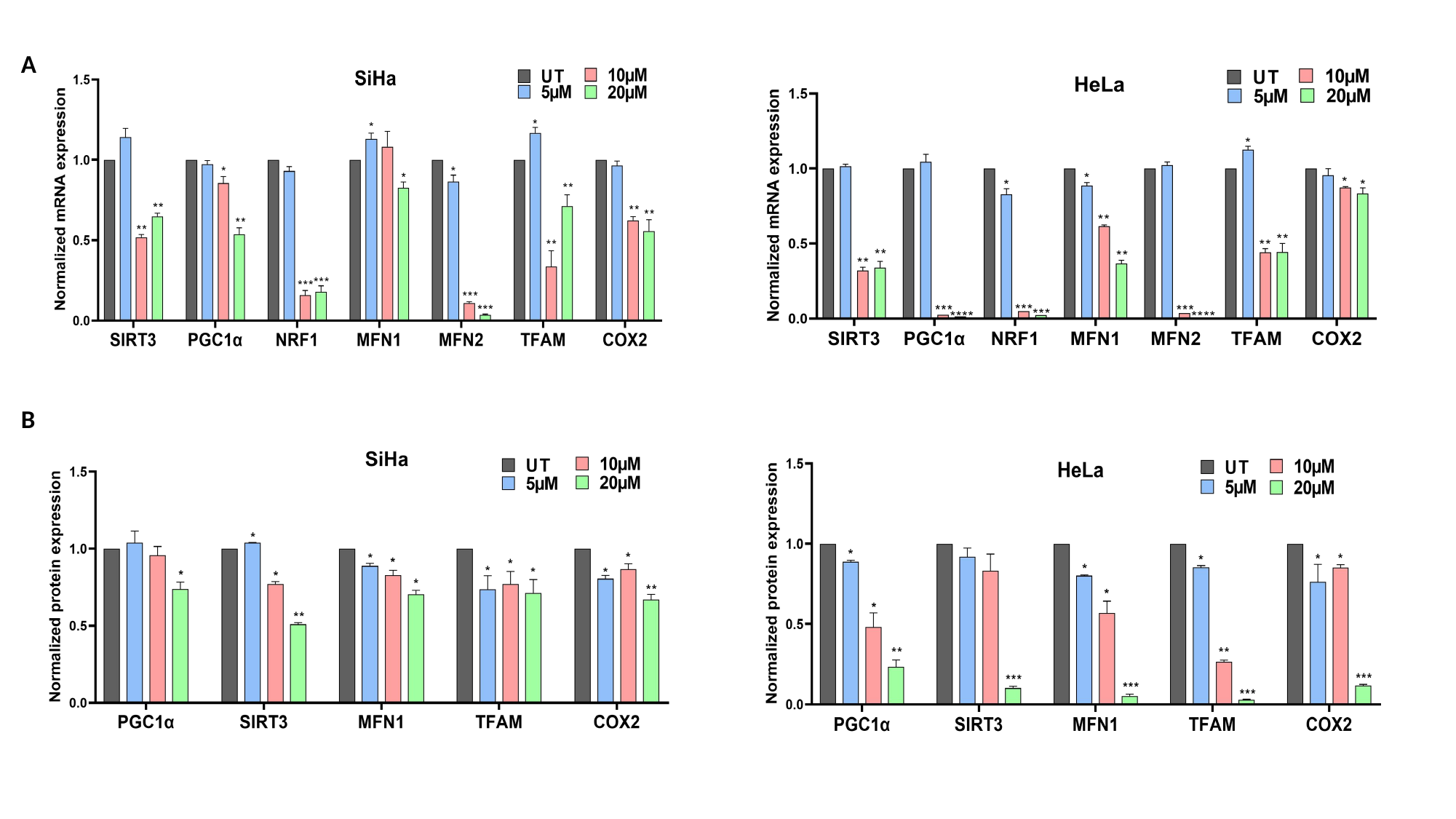

A
B

Supplement: Supplementary file 8 — Supplementary Material 8: Supplementary Fig. 7 Quantification of mitochondrial biogenesis genes at mRNA and Protein level.The bar graph represents the quantification of gene expression at mRNA level in control and PC-treated SiHa and HeLa cells.The bar graph represents the densitometric quantification of gene expression at protein level in control and PC-treated SiHa and HeLa cells *P < 0.05, **P < 0.01, and ***P < 0.001 indicates statistical significance [file 12964_2025_2218_MOESM8_ESM.pptx]
